# Clonal analysis of SepSecS-specific B and T cells in autoimmune hepatitis

**DOI:** 10.1172/JCI183776

**Published:** 2025-01-16

**Authors:** Michael Kramer, Federico Mele, Sandra Jovic, Blanca Maria Fernandez, David Jarrossay, Jun Siong Low, Christiane Sokollik, Magdalena Filipowicz Sinnreich, Sylvie Ferrari-Lacraz, Giorgina Mieli-Vergani, Diego Vergani, Antonio Lanzavecchia, Antonino Cassotta, Benedetta Terziroli Beretta-Piccoli, Federica Sallusto

**Affiliations:** 1 Institute for Research in Biomedicine (IRB), Bellinzona, Switzerland.; 2 Institute of Microbiology, ETH Zurich, Zurich, Switzerland.; 3 Division of Paediatric Gastroenterology, Hepatology and Nutrition, Department of Paediatrics, Inselspital, Bern University Hospital, University of Bern, Bern, Switzerland.; 4 Department of Gastroenterology and Hepatology, Basel University Medical Clinic, Cantonal Hospital Baselland, Liestal, Switzerland.; 5 Department of Biomedicine, University of Basel, Basel, Switzerland.; 6 Transplant Immunology Unit & National Laboratory of Immunogenetics, Division of Nephrology, Department of Diagnostic, University Hospital Geneva, Geneva, Switzerland.; 7 MowatLabs, Faculty of Life Sciences & Medicine, King’s College London, King’s College Hospital, London, United Kingdom.; 8 National Institute of Molecular Genetics, Milano, Italy.; 9 Faculty of Biomedical Sciences, Università della Svizzera italiana, Lugano, Switzerland.; 10 Epatocentro Ticino, Lugano, Switzerland.

**Keywords:** Autoimmunity, Immunology, Hepatitis, Immunoglobulins, T cells

## Abstract

Autoimmune hepatitis (AIH) is a rare chronic inflammatory liver disease characterized by the presence of autoantibodies, including those targeting O-phosphoseryl-tRNA:selenocysteine-tRNA synthase (SepSecS), also known as soluble liver antigen (SLA). Anti-SepSecS antibodies have been associated with a more severe phenotype, suggesting a key role for the SepSecS autoantigen in AIH. To analyze the immune response to SepSecS in patients with AIH at the clonal level, we combined sensitive high-throughput screening assays with the isolation of monoclonal antibodies (mAbs) and T cell clones. The anti-SepSecS mAbs isolated were primarily IgG1, affinity-matured compared with their germline versions, and recognized at least 3 nonoverlapping epitopes. SepSecS-specific CD4^+^ T cell clones were found in patients with AIH who were anti-SLA-positive and anti-SLA-negative,and, to a lesser extent, in patients with non-AIH liver diseases and in healthy individuals. SepSecS-specific T cell clones from patients with AIH produced IFN-γ, IL-4, and IL-10, targeted multiple SepSecS epitopes, and, in one patient, were clonally expanded in both blood and liver biopsy. Finally, SepSecS-specific B cell clones, but not those of unrelated specificities, were able to present soluble SepSecS to specific T cells. Collectively, our study provides the first detailed analysis of B and T cell repertoires targeting SepSecS in patients with AIH, offering a rationale for improved targeted therapies.

## Abstract 2

We characterized the T and B cell response to SepSecS as a specific target of AIH and a model of autoreactivity to a well-defined cytosolic protein.

## Introduction

Autoimmune hepatitis (AIH) is a chronic inflammatory liver disease affecting all ages and ethnicities, characterized by elevation of serum transaminase and IgG levels, positive autoantibodies, and, histologically, by plasma cell rich interface hepatitis (1). The autoimmune nature of AIH is highlighted by several features, including female preponderance, association with extra-hepatic autoimmune diseases, genetic predisposition conferred by specific HLA alleles, and excellent response to immunosuppressive treatment, which needs to be continued life-long in most patients (2). Despite considerable advances in the understanding of AIH pathogenesis, its etiology remains unknown (3).

Two distinct types of AIH are classified based on the presence of specific autoantibodies (1). AIH type 1 (AIH-1) is characterized by the presence of antinuclear antibodies and/or antismooth muscle antibodies. Conversely, AIH type 2 (AIH-2), which is by far less common than AIH-1 and predominantly affects pediatric patients, is defined by the presence of antiliver kidney microsomal type 1 antibodies (targeting cytochrome CYP2D6) and/or antiliver cytosol type 1 antibodies (targeting Formiminotransferase cyclodeaminase). While the HLA allele DRB1*0301 predisposes individuals to both AIH subtypes in all ages, susceptibility to AIH-1 in adults is mainly conferred by the DRB1*0401 allele while susceptibility to AIH-2 in children/adolescents is mainly conferred by the DRB1*0701 allele (4).

Antisoluble liver antigen (SLA) autoantibodies targeting O-phosphoseryl-tRNA:selenocysteine-tRNA synthase (SepSecS), a 56 kDa cytosolic protein of 501 amino acids, are detected in both AIH-1 and AIH-2 and are the only disease-specific AIH-associated autoantibodies (2, 5–8). Additionally, their presence is correlated with more severe disease (6, 9–11). These features suggest a key pathophysiological role of the SepSecS autoantigen in both types of AIH. In the present study, we combined sensitive and high-throughput screening assays with the isolation of monoclonal antibodies (mAbs) and T cell clones to characterize the immune response to SepSecS in patients and individuals in the pathological and healthy control groups for uncovering the pathophysiological role of the SepSecS autoantigen in both types of AIH.

## Results

### Anti-SepSecS serum antibodies and memory B cells measured using newly developed assays.

To explore autoreactive B and T cell immunity in patients with AIH, we collected peripheral blood samples and clinical data from a total of 20 patients (17 patients with a clinical diagnosis of AIH-1 and 3 patients with a clinical diagnosis of AIH-2, Supplemental Table 1; supplemental material available online with this article; https://doi.org/10.1172/JCI183776DS1). Patients were 13–73 years of age (median 51.5) and had different disease duration (range 0–28, median 5.5 years). A total of 7 patients were classified as anti-SLA positive by the clinical laboratory. Seventeen patients were on immunosuppressive treatment at the time of blood collection, of whom 5 had elevated transaminase levels due to an ongoing relapse; 3 patients were studied at presentation, before treatment start (Supplemental Table 1). Blood samples were also obtained from 9 patients with non-AIH liver diseases (pathological control, PC) (Supplemental Table 1) and 12 healthy blood donors (healthy control, HC).

To allow for the sensitive and specific detection of the full spectrum of anti-SepSecS antibodies, we developed 2 different assays based on recombinant SepSecS produced in eukaryotic cells. The first is a flow cytometry–based assay using EXPI-293 cells transfected with a plasmid encoding SepSecS as well as enhanced green fluorescent protein (eGFP). Use of reduced amounts of transfecting reagent and plasmid resulted in approximately 1:1 transfected and nontransfected cells, enabling differentiation between SepSecS-specific antibody binding and unspecific binding (Supplemental Figure 1). The second is an ELISA in which a purified FLAG-tagged SepSecS produced in EXPI-293 cells is coated to high protein-binding plates.

**Figure 1:**
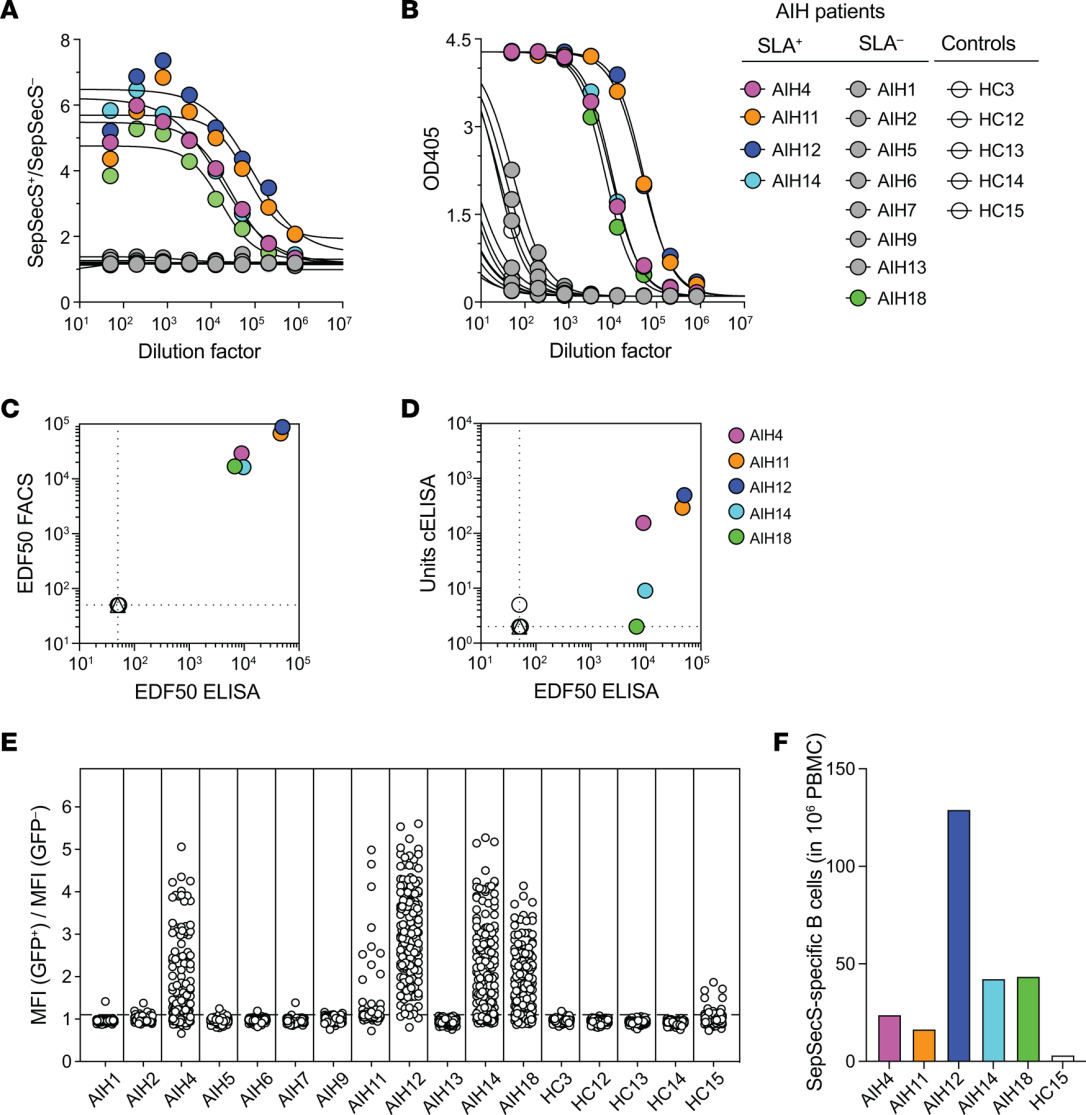
Characterization of anti-SLA positivity in AIH. (**A** and **B**) Measurement of SepSecS-specific IgG in plasma from patients with AIH and individuals in the healthy control group by an in-house–developed flow cytometry assay based on SepSecS/eGFP-transfected EXPI-293 cells (**A**) or by an in-house–developed ELISA using SepSecS produced in EXPI-293 cells (**B**). Note that patient AIH18 was classified anti-SLA negative in the clinical laboratory but is anti-SLA positive in both of our assays. (**C** and **D**) Scatterplots showing the correlation between the EDF_50_ values of SepSecS-specific IgG measured using the flow cytometry assay and ELISA (**C**) and the ELISA and a commercially available ELISA (cELISA) (**D**). (**E**) SepSecS-specific IgG^+^ memory B cells in peripheral blood of patients with AIH and individuals in the healthy control group. PBMCs (3 × 10^4^; AIH11: 2 × 10^4^) were plated in 192 replicate wells (AIH11, 96) and stimulated with IL-2 and the TLR 7/8 agonist R848. After 12 days, the supernatant of each well was screened for the presence of secreted SepSecS-specific IgG using the flow cytometry assay. Reported is the mean fluorescence intensity (MFI) GFP-SepSecS^+^ / MFI GFP-SepSecS^–^ ratios for each individual supernatant (positive > 1.1). (**F**) Number of SepSecS-specific IgG^+^ memory B cells in 1 × 10^6^ PBMCs in patients with AIH and individulals in the healthy control group with positive cultures in the analysis in **E** and calculated according to the Poisson distribution.

The two in house-developed assays were used in parallel and together with a commercially available ELISA (Euroimmun) to measure SepSecS-specific IgG in the plasma of 12 patients with AIH (10 AIH-1 and 2 AIH-2) and of 5 individuals in the HC group. As shown by the binding curves (Figure 1, A and B), both the flow cytometry assay and the ELISA detected SepSecS-specific IgG in the plasma of the patients classified as anti-SLA positive (AIH4, AIH11, AIH12, AIH14). Both methods, however, also allowed the detection of anti-SepSecS IgG in patient AIH18, who was classified as anti-SLA negative by the clinical laboratory using a commercial assay. The effective dilution factor at 50% maximum (EDF_50_) values calculated from the binding curves of the two assays were comparable (Figure 1C and Supplemental Table 2). Unspecific binding was only detected at very high plasma concentrations in the ELISA and at intermediate concentrations in the flow cytometry assay, where it could easily be discerned by comparing staining of transfected and nontransfected cells. The sensitivity of the newly developed ELISA and flow cytometry assay was higher compared with a commercially available ELISA (Figure 1D and Supplemental Table 2) and therefore suitable for high-throughput screening of the antibody response in AIH.

To investigate the B cell response to SepSecS in patients with AIH and individuals in the control groups, we measured the frequency of SepSecS-specific circulating memory IgG^+^ B cells. Peripheral blood mononuclear cells (PBMCs), obtained from the same blood sample in which antibodies were measured, were stimulated with IL-2 and the Toll-like receptor 7/8 agonist R848 for 12 days, as previously described (12, 13). The flow cytometry–based assay was then used to detect SepSecS-specific IgG in the culture supernatants. Memory B cells secreting SepSecS-specific IgG antibodies were found in the 4 anti-SLA–positive patients (AIH4, AIH11, AIH12, AIH14) as well as in patient AIH18 that was classified as anti-SLA positive according to our assays (Figure 1, E and F). No SepSecS-specific IgG^+^ B cells were found in the peripheral blood of anti-SLA negative patients and of healthy controls, with the exception of HC15 who harbored few SepSecS-specific IgG^+^ B cells producing antibodies with very weak binding (Figure 1, E and F). Taken together, these findings indicate that the new high-throughput assays based on SepSecS expressed on eukarytotic cells are suitable for the detection of specific antibodies in the serum and culture supernatants.

### Anti-SepSecS mAbs are affinity matured and recognize 3 distinct sites.

**Figure 2:**
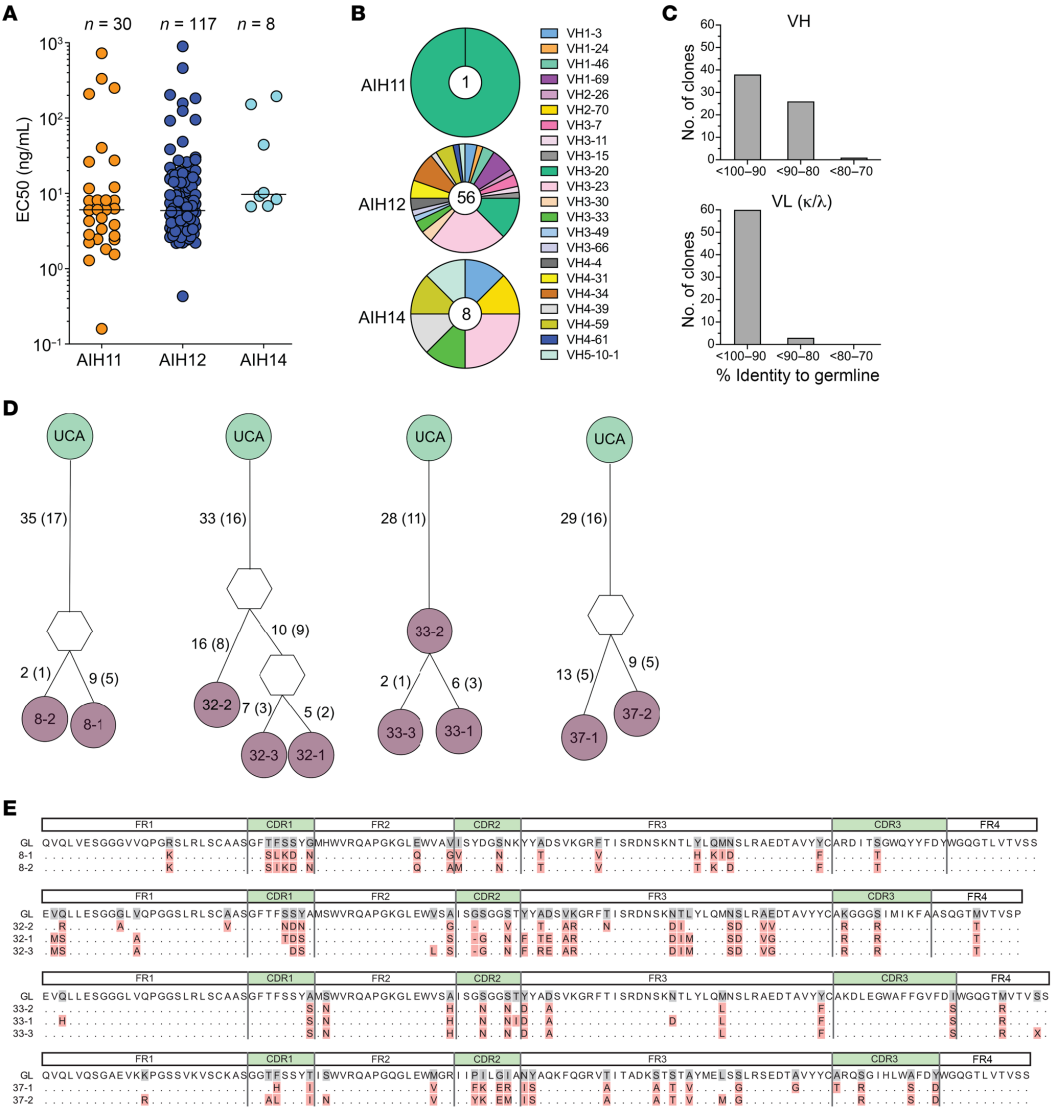
SepSecS-specific antibodies in patients with AIH are polyclonal and affinity matured. (**A**) EC_50_ values of 155 anti-SepSecS mAbs isolated from EBV-immortalized IgG^+^ memory B cell clones from patients AIH11, AIH12, and AIH14, as calculated from curves reported in Supplemental Figure 3. (**B**) VH gene usage of sequenced SepSecS-specific B cell clones. Slices in the chart represent different VH genes, and their size is proportional to the number of clones using that particular gene. The total number of sequenced clones from each patient is reported at the center of the pie charts. (**C**) Percentages of sequenced SepSecS-specific B cell clones with variable identity to the germline VH and VL genes. (**D**) Genealogical trees of 4 B cell clonal families in patient AIH12. The number of somatic mutations at nucleotide and amino acid level (in parenthesis) is indicated on individual branches of the trees. Unmutated common ancestors (UCAs) are in green, intermediary development steps are in white, and sequenced clones are in purple. (**E**) Sequence alignments show heavy chain amino acid mutations of sequenced clones from the respective UCAs.

To investigate the properties of SepSecS-specific antibodies at monoclonal resolution, we generated B cell clones from CD19^+^IgG^+^ memory B cells isolated from PBMCs (Supplemental Figure 2) of 3 anti-SLA-positive patients (AIH11, AIH12, and AIH14), using an established protocol (14). By using the flow cytometry assay to screen the mAbs in the culture supernatants, we identified 30, 117, and 8 SepSecS-specific B cell clones from patients AIH11, AIH12, and AIH14, respectively. We quantified IgG in the culture supernatants and obtained binding curves from all 155 mAbs from which the EC_50_ was calculated (Figure 2A and Supplemental Figure 3). Notably, most SepSecS-specific autoreactive mAbs (108 out of 155; 70%) showed high affinity, with EC_50_ values between 1 and 10 ng/mL, mAbs obtained from different patients displaying comparable mean EC_50_ values.

Sequencing of immunoglobulin genes of 65 B cell clones from patients AIH11, AIH12, and AIH14, showed that 56 antibodies were IgG1, 8 were IgG2, and 1 was IgG3. These antibodies used diverse VH genes and displayed moderate levels of somatic mutations (Figure 2, B and C and Supplemental Table 3). The large number of B cell clones sequenced from patient AIH12 revealed that 10 of the 56 B cell clones belonged to 4 clonal families, each with identical V(D)J usage but different somatic mutations that were expanded in vivo (Figure 2, D and E).

**Figure 3:**
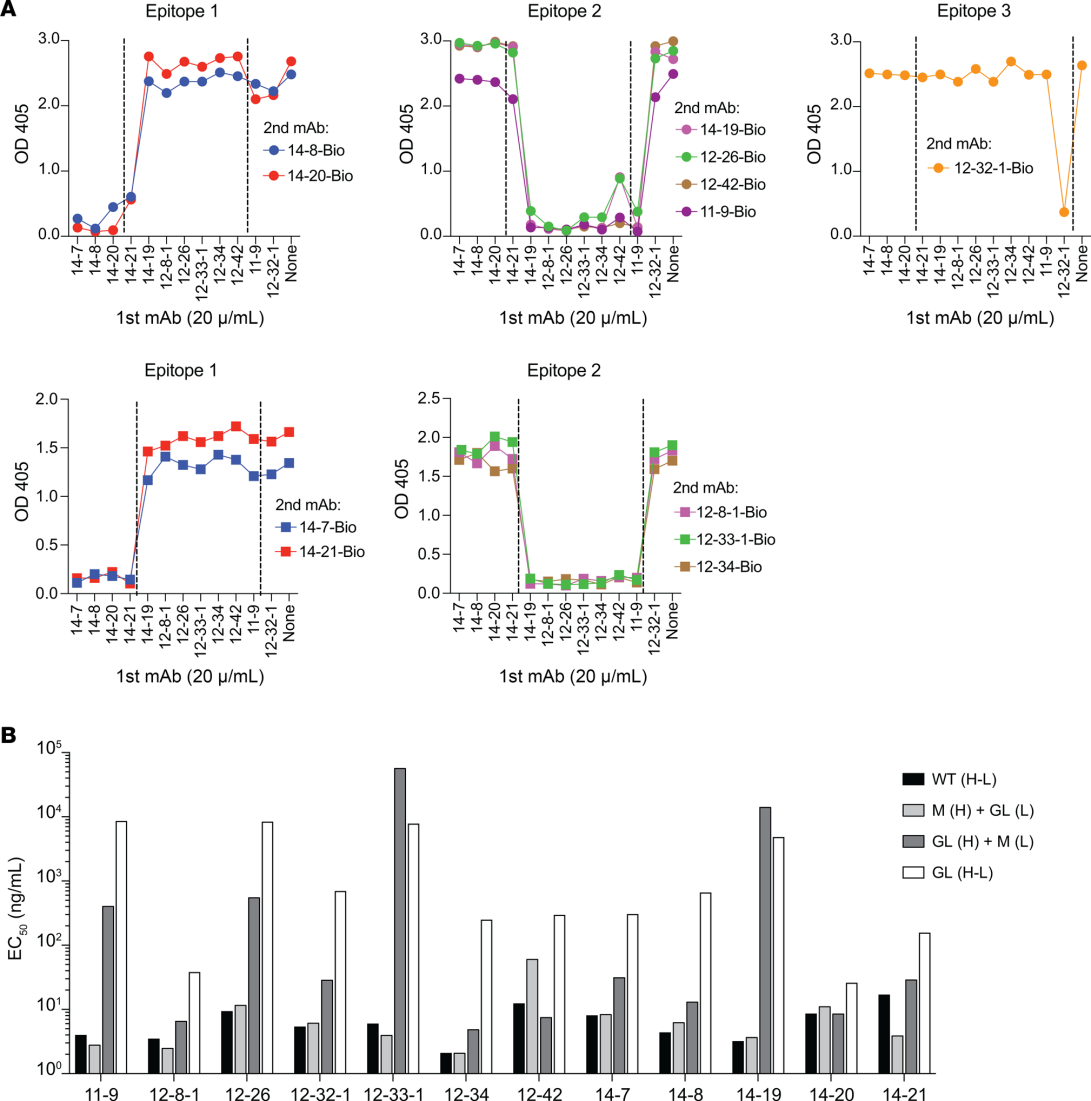
SepSecS-specific mAb recognize different epitopes and acquire high affinity through somatic mutations. (**A**) Competition-ELISA identifying 3 groups of recombinantly produced mAbs with different binding regions. ELISA-plates were coated for 24 hours at 4°C with 5 μg/mL SepSecS. First, mAbs were added in excess (20 μg/mL) to block mAb-specific epitopes completely. Biotinylated mAbs were added as second mAbs, and binding was assessed using streptavidin-HRP. The 12 recombinant mAbs were analyzed in 2 independent experiments of 7 (upper 3 plots) and 5 mAbs (lower 2 plots). MAb codes start with number of the patient with AIH from whom the mAb is derived. (**B**) EC_50_ values of SepSecS binding of recombinantly produced mAbs carrying different combinations of mutated (M) and germline (GL) heavy and light chain of the indicated mAbs as measured by flow cytometry assay. MAb codes start with number of the patient with AIH from whom the mAb is derived.

Twelve mAbs (1, 6, and 5 from patients AIH11, AIH12, and AIH14, respectively) were selected for in-depth studies and produced recombinantly. Binding competition experiments with the purified mAbs revealed that the 12 mAbs bind to 3 distinct regions, with epitopes in region 2 targeted by mAbs from all 3 patients (Figure 3A).

To investigate the role of somatic mutations in antigen binding, we produced the 4 possible combinations of mutated (M) and germline (GL) heavy and light chains for each of the 12 mAbs and assessed the EC_50_ values for each of the 48 combinations (Figure 3B). All the M_heavy_+M_light_ (defined as WT) mAbs bound SepSecS with increased affinity over their respective GL_heavy_+GL_light_ combinations, indicating that somatic mutations contribute to affinity maturation. However, the extent of affinity increase varied between different mAbs. In some cases, the GL version already bound SepSecS with moderate affinity (EC_50_ of 2 GL mAbs: 10–100 ng/mL and of 6 GL mAbs: 100–1000 ng/mL). Finally, the comparison of EC_50_ values of M_heavy_+GL_light_ and GL_heavy_+M_light_ combinations revealed a higher influence of heavy chain mutations than light chain mutations on affinity maturation (Figure 3B). Collectively, these findings demonstrate that SepSecS-specific antibodies are polyclonal, use diverse V(D)J genes, and acquire high affinity binding through somatic mutations.

### High CD4^+^ T cell proliferative response to SepSecS in patients with AIH.

In parallel with the investigations of the antibody response to SepSecS, we investigated the CD4^+^ T cell response to the same antigen. This analysis was performed on all patients with AIH and patients in the PC group included in the study (Supplemental Table 1) and in 12 individuals in the HC group. We genotyped the 20 patients with AIH and found that 16 carried at least 1 AIH-predisposing HLA allele (Supplemental Table 1).

**Figure 4:**
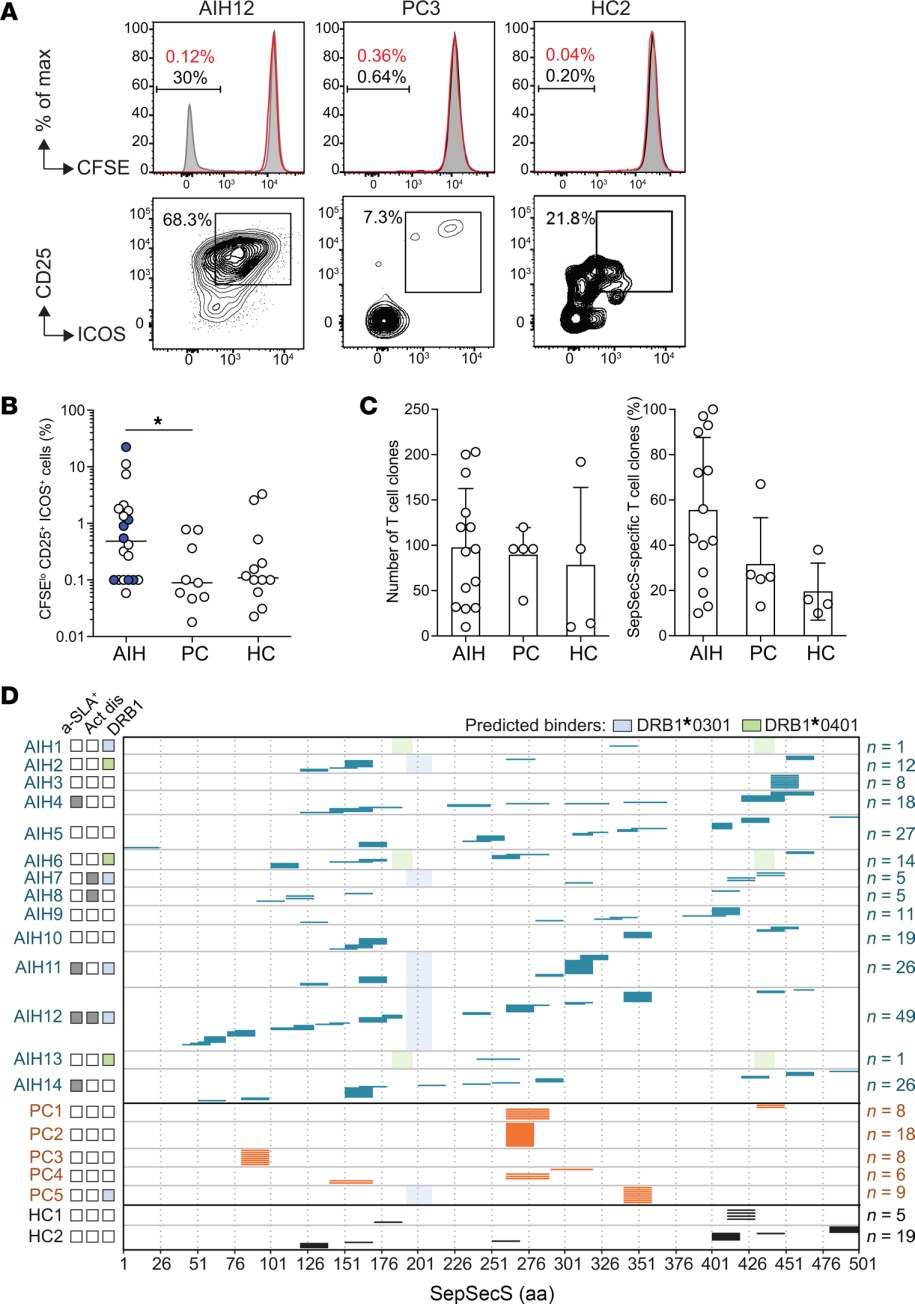
SepSecS proliferative response of memory CD4^+^ T cells in the blood of patients with AIH and individuals in the control groups. Total memory CD4^+^ T cells from the blood of patients with AIH, patients with non-AIH liver diseases in the PC group and individuals in the HC group were labeled with CFSE and stimulated with autologous monocytes in the presence or absence of the SepSecS peptide pool. On day 7, cells were collected and stained with anti–CD25-PE and anti–ICOS-APC mouse mAbs. (**A**) Histograms represent CFSE profile and contour plots represent CD25 and ICOS staining on gated CFSE^lo^ cells from AIH12 (anti-SLA positive), PC3 and HC2. Red lines, no SepSecS; gray lines, SepSecSpp. (**B**) Pooled data from all study participants shown as the percentage of CFSE^lo^CD25^+^ICOS^+^ cells in the SepSecS stimulated cultures. Lines represent the median values. Blue dots represent anti-SLA positive patients with AIH. *P < 0.05 when determined by 2-tailed Mann-Whitney test and < 0.01 when determined by Spearman Partial correlation considering age as a covariable. (**C**) Number of total CD4^+^ T cell clones (left) isolated by limiting dilution from sorted CFSE^lo^CD25^+^ICOS^+^ cells from ex vivo cultures of memory CD4^+^ T cells from patients with AIH, and individuals in the PC and HC groups. The right plot shows the frequency of SepSecS-specific CD4^+^ T cell clones among the isolated clones for each donor. Specificity was tested by stimulation with irradiated autologous B cells untreated or pulsed with SepSecS peptide pool. Proliferation was measured on day 3 after a 16 hour incubation with 1 μCi/mL methyl-^3^H-thymidine. (**D**) Epitope mapping of SepSecS-specific CD4^+^ T cell clones from patients with AIH and individuals in the control groups. Epitopes were identified by stimulating the clones with individual SepSecS peptides. Patients with AIH are represented in blue, patients in the PC group are in orange and individuals in the HC group are in black. Predicted binding peptides are shown as shaded blue (DRB1*03:01) and shaded green (DRB1*0401) squares in patients carrying the AIH predisposing HLA alleles DRB1*0301 and DRB1*0401, respectively. Anti-SLA status and disease activity are shown on the left-hand side; number of CD4^+^ T cell autoreactive clones are shown on the right-hand side.

Total memory CD4^+^ T cells were isolated by cell sorting from PBMC samples of patients and controls (Supplemental Figure 2). The sorted total memory CD4^+^ T cells were labelled with carboxyfluorescein succinimidyl ester (CFSE), and then stimulated with autologous monocytes, which were either untreated or pulsed with a pool of 20-mer overlapping peptides (50 peptides) spanning the entire sequence of the SepSecS protein (SepSecSpp). When assessed on day 7, the T cells of patients with AIH had, on average, a higher proliferative response compared with individuals in the control groups, as demonstrated by a higher frequency of CFSE^lo^CD25^+^ICOS^+^ cells, but SepSecSpp-induced T cell proliferation was also detected in some of the individuals in the PC and HC groups (Figure 4, A and B). The strongest proliferative T cell responses were detected in patient AIH12, who was anti-SLA positive and had elevated transaminase levels, and in patients AIH3 and AIH5, who were anti-SLA-negative and had normal transaminase levels (Supplemental Table 4).

To further characterize SepSecS autoreactive CD4^+^ T cells from the blood of patients with AIH and individuals in the control groups, the CFSE^lo^CD25^+^ICOS^+^ cell fraction was sorted and cells were cloned by limiting dilution. A total of 1,364 CD4^+^ T cell clones were isolated from 14 patients (4 anti-SLA positive, 10 anti-SLA negative, including 2 with AIH-2). These clones were screened for reactivity against SepSecSpp using autologous Epstein Barr virus–immortalized (EBV-immortalized) B cells as antigen-presenting cells (APCs) and found to be mostly SepSecSpp-specific. A total of 759 CD4^+^ T cell clones were isolated from individuals in the control groups (5 PCs and 4 HCs). Interestingly, however, the mean percentage of SepSecS-specific clones, as defined by a stimulation index of greater-than 5, was significantly lower in individuals in the control groups compared with patients with AIH (31.6% versus 55.4% for PCs, and 19.5% versus 55.4% for HCs, *P* < 0.001) (Figure 4C and Supplemental Table 4), suggesting that recruitment of bystander T cells may contribute to the proliferative response of controls. Collectively, the results obtained using ex vivo antigenic stimulation and single T cell cloning show that, although SepSecS-specific memory CD4^+^ T cells are present in both patients with AIH and individuals in the control groups, the response is significantly higher in patients with AIH, irrespective of anti-SLA positivity and of disease activity.

### SepSecS-reactive CD4^+^ T cells target a diverse set of epitopes and produce multiple cytokines.

The fine specificity of 222 SepSecS-specific CD4^+^ T cell clones isolated from 14 patients with AIH and of 73 SepSecS-specific CD4^+^ T cell clones isolated from 7 individuals in the control groups was determined by epitope mapping using individual peptides spanning the entire SepSecS protein (Figure 4D). Alignment of all mapped epitopes showed that patients recognized multiple epitopes spanning the entire SepSecS sequence. In particular, there was a trend for recognition of multiple epitopes in T cells from patients with AIH who were anti-SLA-positive compared with anti-SLA-negative and especially individuals in the PC and HC groups (Figure 4D). Furthermore, the amino acid regions 151–170 and 161–180 were the most frequently targeted regions by the T cell clones from patients with AIH, being recognized by 22 and 25 out of 222 clones and by 6 and 7 out of 14 patients, respectively, including all anti-SLA–positive patients (Figure 4D and Supplemental Figure 4). Conversely, the sequence between amino acids 261 and 280 was the most frequently targeted region by T cell clones isolated from individuals in the control groups, being recognized by 27 out of 73 clones and by 3 out of 7 individuals (Figure 4D and Supplemental Figure 4). Of note, in patients carrying the AIH-predisposing HLA alleles DRB1*0301 and DRB1*0401, the identified SepSecS peptides were not predicted to be strong binders using the NetMHCIIpan tool (Figure 4D).

Cytokine concentrations in the culture supernatants of antigen-stimulated SepSecS-specific CD4^+^ T cell clones were measured using a Luminex bead-based assay, revealing that the vast majority of clones from both patients and individuals in the control groups coproduced IFN-γ, IL-4, and, notably, the antiinflammatory cytokine IL-10 (Supplemental Figure 5, A–D and Supplemental Table 5).

In conclusion, the recognition of many different epitopes by SepSecS-reactive CD4^+^ T cell clones indicates a polyclonal and multifunctional response of CD4^+^ T cells to SepSecS in both patients and individuals in the control groups, with an immunodominant region between amino acids 151 and 180 targeted by the majority of patients with AIH.

### SepSecS-specific CD4^+^ T cells are clonally expanded in blood and liver.

**Figure 5:**
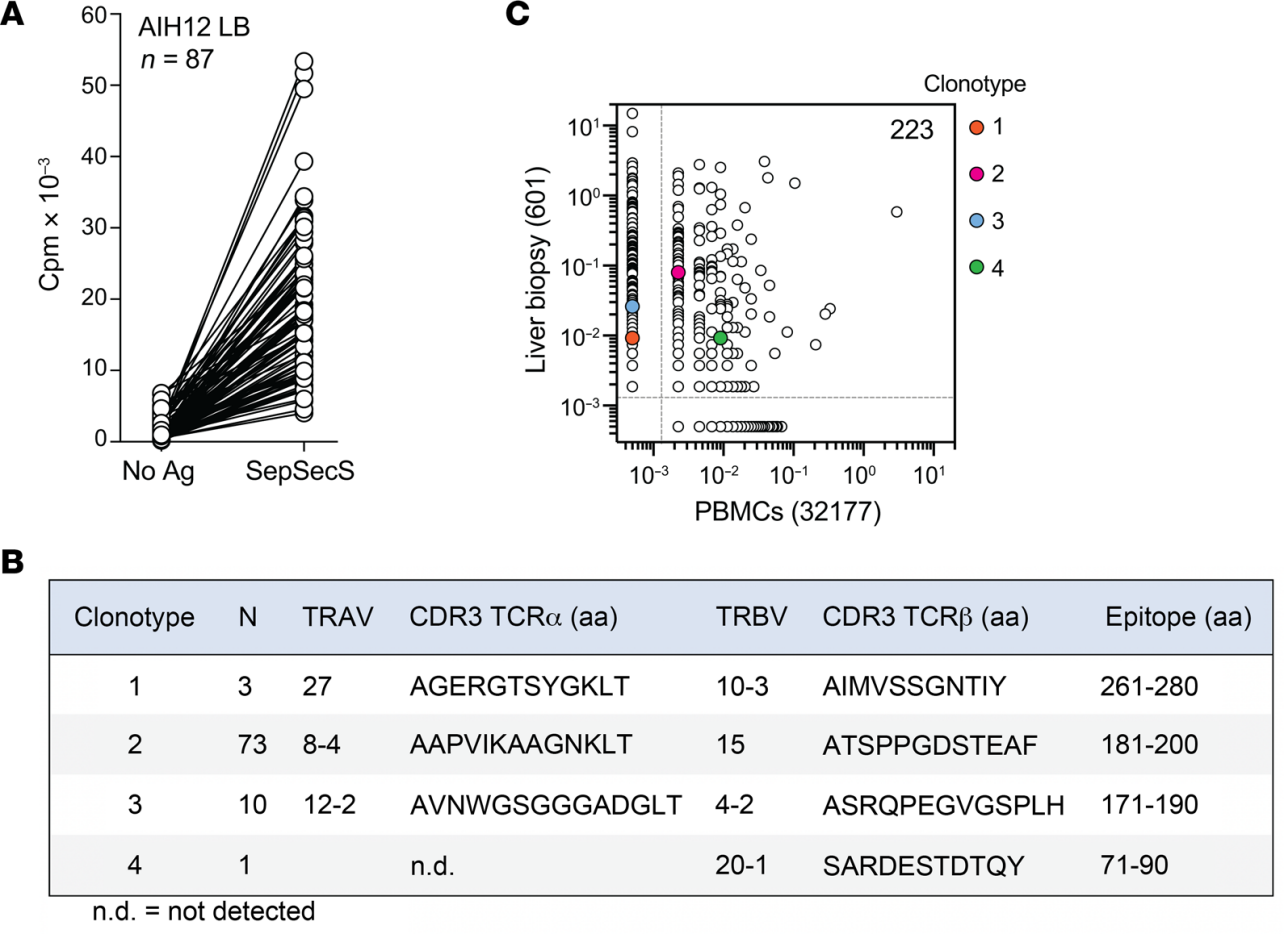
SepSecS-specific CD4^+^ T cells are clonally expanded in the blood and liver of patient AIH12. (**A**) Proliferative response to SepSecS of CD4^+^ T cell clones from the liver biopsy of patient AIH12. Cpm, counts per minute. Autologous EBV-B cells were either left untreated (No Ag) or pulsed with SepSecSpp. (**B**) TCR Vα and TCR Vβ chain sequencing of the 87 SepSecS-specific CD4^+^ T cell clones identify 4 unique clonotypes. Reported are the T cell receptor α variable (TRAV) gene and the T cell receptor β variable (TRBV) gene used as well as the CDR3 amino acid (aa) sequences of the TCRα and TCRβ chains. The number of identical sequences per each clone and the SepSecS epitopes, as identified by epitope mapping, are shown. aa, amino acids. (**C**) Pairwise comparison of TCR Vβ clonotype frequency distribution in samples of total memory CD4^+^ T cells sorted from PBMCs (x-axis) and in vitro-expanded total memory CD4^+^ T cells from a liver biopsy taken on the same day as the PBMCs (y-axis). The 4 SepSecS-specific clonotypes, which were identified by TCR Vβ chain sequencing of the 87 CD4^+^ T cell clones isolated from liver-infiltrating T cells are highlighted in color.

To investigate whether autoreactive T cells could be found also in the target organ, fresh liver biopsies were obtained from 5 patients (AIH2, AIH12, AIH16, AIH19, and AIH20). Infiltrating T cells were isolated and polyclonally expanded in vitro. CD4^+^ T cells were then isolated, labeled with CFSE, and stimulated with irradiated autologous B cells, which were either untreated or pulsed with the SepSecSpp. CFSE^lo^CD25^+^ proliferating T cells were only detected in samples from patients AIH12 and AIH16 (Supplemental Table 4). These cells were sorted and cloned by limiting dilution. Out of 130 CD4^+^ T cell clones from patient AIH12, 87 were SepSecS specific, while no specific T cell clones were found among the 41 clones from patient AIH16 (Figure 5A and Supplemental Table 4). Sequencing of the T cell receptor (TCR) β-chain and α-chain variable regions of the 87 liver-derived SepSecS-specific CD4^+^ T cell clones from patient AIH12 identified 4 unique clonotypes (Figure 5B). Epitope mapping using the individual peptides spanning SepSecS, identified 4 distinct regions targeted by 73 (amino acids 181–200), 10 (amino acids 171–190), 3 (amino acids 261–280) and 1 (amino acids 71–90) clones (Figure 5B). These epitopes partially matched the epitopes identified in T cell clones isolated from the blood of the same patient. Similar to the clones isolated from blood, the cytokine production profile of the liver-infiltrating T cell clones was marked by coproduction of IFN-γ and IL-4, as well as IL-10 (Supplemental Figure 5E and Supplemental Table 5). Notably, the liver-infiltrating T cell clones produced significantly higher amounts of these cytokines compared with circulating T cell clones, suggesting a more pronounced effector function within the tissue environment.

To investigate whether the TCR clonotypes detected in the liver biopsy were present in circulating T cells, we performed TCR Vβ CDR3 sequencing of total memory CD4^+^ T cells obtained from a blood sample collected at the time of the liver biopsy as well as of in vitro–expanded CD4^+^ T cells obtained from the liver biopsy. We detected 32,177 clonotypes in 5 × 10^5^ total circulating memory T cells and 601 clonotypes in 5 × 10^5^ in vitro–expanded T cells from liver biopsy (Figure 5C). Of the latter, 223 (corresponding to 37% of unique clonotypes) were also found in peripheral blood memory T cells. Notably, 2 of the 4 clonotypes identified by single T cell cloning of liver-infiltrating T cells were also identified in blood (Figure 5C). Collectively, we found that SepSecS-specific CD4^+^ T cell clones can be identified among liver-infiltrating T cells and at least partially overlap with the SepSecS-specific CD4^+^ T cell clonotypes present in the blood of the same patient.

### SepSecS-specific B cells effectively present the autoantigen to specific CD4^+^ T cells.

**Figure 6:**
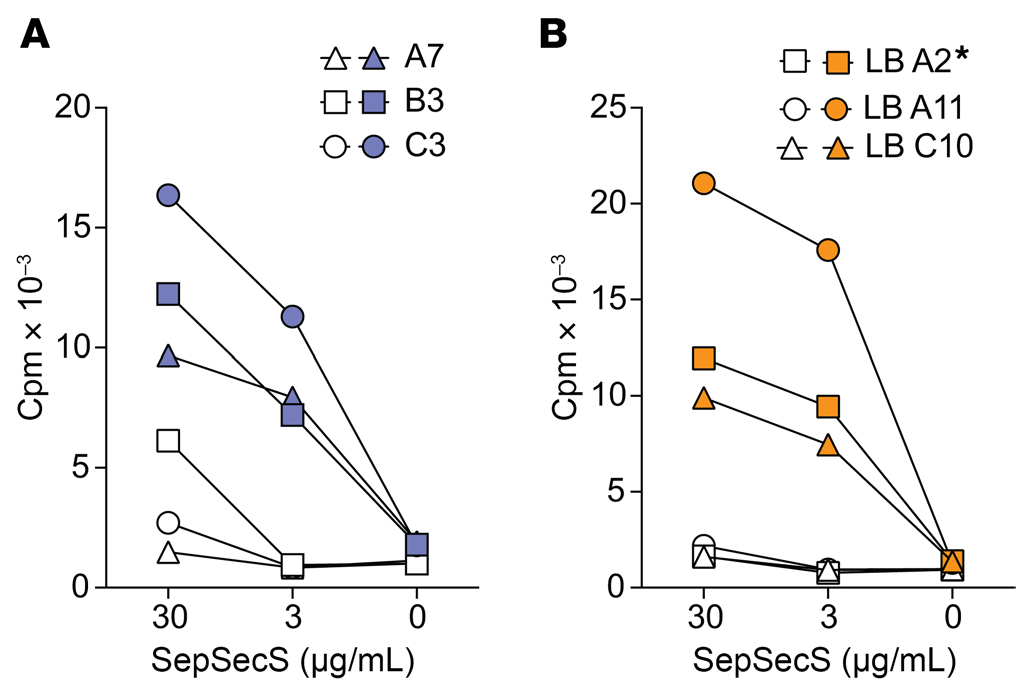
Specific B cells effectively present SepSecS protein to specific CD4^+^ T cells. (**A** and **B**) Proliferative response of CD4^+^ T cell clones derived from blood (**A**) or liver (**B**) of patient AIH12 to different concentrations of SepSecS protein. The T cell clones targeted different SepSecS epitopes (C3 and A7: aa 341-370; B3: aa 71-90; LB A11 and LB C10: 181-200; LB A2*: 261-280). A SepSecS-specific (blue or orange symbols) EBV-B cell clone and an EBV-B cell clone of unrelated-specificity (white symbols) derived from the same patient were used as APCs. Cpm, counts per minute.

Antigen-specific B cells are known to be effective APCs and could play a role in activating T cells in AIH (15–17). To investigate the ability of SepSecS-specific B cells to drive the proliferation of SepSecS-specific CD4^+^ T cells, we selected a SepSecS-specific- (clone 56; EC_50_ 16.35 ng/mL) and a SepSecS-unreactive B cell clone from AIH12. We then compared the proliferative response of SepSecS-specific CD4^+^ T cell clones to recombinant SepSecS protein presented by the 2 populations of EBV-B cells (Figure 6). All SepSecS-specific CD4^+^ T cell clones (3 derived from blood and 3 from liver) showed a strong proliferative response to SepSecS presented by SepSecS-specific B cells, though failed to proliferate in response to unreactive B cells, even in the presence of the highest SepSecS dose. The T cell clones recognized 4 distinct epitopes, and T cell clones specific to the same epitope exhibited similar functional responses. No clear differences between the response of blood- and liver-derived clones were observed. Collectively, these findings show that SepSecS-specific B cells can effectively process and present the self antigen to CD4^+^ T cells of cognate antigen specificity.

## Discussion

In this study we characterized the immune response to SepSecS as a specific target of AIH and a model of autoreactivity to a well-defined cytosolic protein. To this aim, we developed serological assays to measure serum and secreted antibodies using high-throughput and -sensitivity techniques and isolated human mAbs and T cell clones. SepSecS-specific serum antibodies and memory B cells were found in all patients with anti-SLA–positive AIH and in an additional patient who was scored anti-SLA-negative in clinical laboratory, but not in other patients who were anti-SLA-negative or in individuals in the control groups. The mAbs isolated from memory B cells were class-switched, primarily IgG1, somatically mutated, and bound to SepSecS with high affinity. Although we have not determined the precise epitopes of the anti-SepSecS mAbs isolated, competition experiments indicated that 11 out of 12 mAbs bind to a single site out of 3 characterized, likely representing a key target of anti-SepSecS reactivity. This finding is consistent with previous studies identifying a region near the carboxy-terminus of the SepSecS as the sole or main target of serum antibodies in a large number of patients with AIH (7, 18).

An interesting finding of our study is that the unmutated precursors of SepSecS antibodies were self reactive, since they bound, albeit with lower affinity, to SepSecS. This in contrast with previous studies on autoantibodies to DNA, desmogleins, and GM-CSF where self reactivity was shown to be generated through somatic mutations (19–21). Thus, while somatic hypermutation contributed to affinity maturation, the naive B cells in patients with AIH were intrinsically autoreactive, a fact that may be explained by the cytosolic location of SepSecS that may prevent induction of B cell tolerance (22, 23). Despite being intracellular, SepSecS may become accessible to the immune system due to the liver’s unique anatomical location, which continuously exposes hepatocytes to a variety of toxins, xenobiotics, gut-derived microbial products, and hepatotropic viruses, with consequent release of intracellular proteins (24).

The T cell response to SepSecS was studied using a sensitive method based on stimulation of purified memory T cells with autologous monocytes pulsed with a pool of overlapping peptides (SepSecSpp) followed by the isolation and characterization of T cell clones. Interestingly, while specific antibodies and memory B cells were only found in a subgroup of patients with AIH, memory T cells proliferating in response to SepSecSpp were detected not only in anti-SLA–positive and anti-SLA–negative patients with AIH, but also, albeit at lower frequency, in patients in the PC group and individuals in the HC group. In addition, while most T cell clones isolated from proliferating T cells of patients with AIH were SepSecSpp specific, only a small fraction of T cell clones from controls responded to SepSecSpp stimulation. Furthermore, T cell clones isolated from patients with AIH, in particular those who were anti-SLA-positive, recognized multiple epitopes spanning the whole protein sequence. In contrast, T cell clones isolated from controls were found to recognize a single or a few peptides. Collectively, these findings suggest that, in quantitative and qualitative terms, the response to SepSecS is higher in patients with AIH, which is in line with a previous report based on ELISpot analysis showing a positive association between the breadth of recognition and liver damage (25). Importantly, in this study we provide evidence that some of the SepSecS-specific T cell clonotypes isolated from a patient with AIH are found expanded in blood and can be present in the liver, supporting their role in pathology. A general limitation of our and similar epitope mapping studies performed with peptides is that only a small fraction of all potentially HLA-binding peptides is generated by antigen processing of native antigens (26–29).

In previous studies, autoreactive SepSecS-specific CD4^+^ T cells were only detected in patients who were anti-SLA positive (30) whereas in our study, these autoreactive T cells were also found in patients with AIH who were anti-SLA negative and, at a significantly lower frequency, in individuals in the control groups. The isolation of SepSecS-specific T cells from controls may be related to the exclusion of Treg cells in our culture conditions, a procedure that facilitates the analysis of weak T cell responses or of low-affinity cross-reactive T cells (31). Moreover, recent studies have provided evidence that frequency of T cells recognizing self and nonself antigens is comparable (32–33) and that autoreactive T cells specific for a set of tissue-restricted self antigens are controlled by specific Tregs rather than central deletion (33). Of note, a numerical and functional impairment of Tregs has been unambiguously demonstrated in AIH (34–37), suggesting that failure of regulatory mechanisms may underlie the development of overt autoimmunity in AIH. Intriguingly, most CD4^+^ T cell clones isolated in our study are multifunctional, producing both IFN-γ and IL-10, suggesting they can exert proinflammatory but also immunosuppressive or protective functions. It is also possible that some of the anti-SepSecS–reactive T cells may originate from cells with regulatory properties, considering that Tregs can acquire effector functions in an inflammatory environment (38). However, it is important to note that the cytokines were measured in T cell clones expanded in vitro, which may not fully represent the functional behavior of T cells in the physiological context.

The isolation of B and T cell clones was instrumental to demonstrate the antigen-dependent interaction that may underline clonal expansion and autoantibody production. These findings also suggest that autoreactive memory B cells may rely on their high antigen presenting functions to exert pathogenicity. A key role of B cells in the pathogenesis of AIH is supported by the clinical observation that B cell depletion with rituximab, an anti-CD20 mAb, or belimumab, an anti-BAFF mAb, is an effective third-line therapy for patients who are difficult to treat (39–43). Moreover, in a mouse model of type 2 AIH, depletion of B cells was shown to induce remission of liver disease through reduced autoantigen presentation to T cells (16). Together with our data that SepSecS-specific B cells are found only in patients who are anti-SLA positive while SepSecS-specific CD4^+^ T cells are present in patients with AIH irrespective of anti-SLA status and also in individuals in the control groups, these findings highlight a key pathophysiological role for B cells in AIH, offering a rationale for the design of improved, targeted therapies to treat AIH.

Our findings form the basis for further investigations on the role of anti-SepSecS reactivity, which is associated with a severe AIH clinical phenotype. Extending the identification of TCRs and target epitopes of SepSecS-reactive T cells from a larger cohort of patients will provide further validation and insights into the immunodominant region identified in this study and its relevance in AIH. The data will also enable the establishment of novel animal models investigating the pathogenic pathways in both types of AIH, which are, to date, limited to the much less frequent type 2 AIH, where target autoantigens and immunodominant epitopes are better characterized.

## Methods

### Sex as a biological variable.

The study includes both men and women in both the patient and control groups, with a predominance of female participants, as AIH affects mainly females.

### Patients and sample collection.

Blood samples were collected from 20 patients with AIH (17 AIH-1, 3 AIH-2), of whom 7 were anti-SLA positive according to the clinical laboratory using commercially available ELISA or dot-blots, depending on the hospital’s practices. Patient AIH18 was classified as anti-SLA negative in the clinical laboratory using the Euroimmun commercial ELISA (Cat. EA 1302-9601 G), but was anti-SLA positive using our assays. Seventeen patients were on immunosuppressive treatment, of whom 5 had a relapse at time of blood collection; 3 patients (AIH16, AIH19, and AIH20) were investigated at presentation before treatment start; 1 patient (AIH12) had undergone liver transplantation for severe AIH and experienced posttransplant AIH recurrence at the time of blood collection. The median age was 51.5 years (range 13–73), 17 participants were female, and the median disease duration was 5.5 years (range 0–28 years). Liver biopsies were obtained from patients AIH2, AIH12, AIH16, AIH19, and AIH20 with active disease. As controls, we obtained blood samples from 15 healthy participants (blood donors) and from 9 patients with non-AIH liver diseases, of whom 7 had primary biliary cholangitis. HLA-A/B/C/DRB1 and DQB1 typing of the patients was performed either by sequence-specific oligonucleotide (SSO) DNA-typing (LABType HD) or sequence-specific primer (SSP) typing (Olerup SSP).

### Cell culture.

EXPI293F cells (Gibco) were cultured in Expi293 Expression Medium according to the manufacturer’s instructions. Other cells were cultured at 37°C, 5% CO_2_ using, if not mentioned otherwise, complete medium (RPMI 1640 medium (cat. 31870-025), 2 mM glutamine (cat. 35050-038), 1% (vol/vol) nonessential amino acids (cat. 11140050, 1% (vol/vol) sodium pyruvate (cat. 11360-039), PenStrep (50 U/mL penicillin, 50 μg/mL streptomycin, cat. 15070-063), Kanamycin (50 U/mL, cat. 15160-047), 0.1% β-mercaptoethanol (cat. 31350- 010) (all from Gibco), 10% FBS (HyClone, characterized, GE Healthcare Life Science). For culturing of T cells 5% heat-inactivated human serum (Swiss Red Cross) was used instead of FBS. For pure B cell cultures, 30 μg/mL Transferrin (LubioScience, cat. 0905-100) were added to complete medium. All cells were routinely tested for mycoplasma contamination.

### Human sample processing and sorting.

Blood samples were processed to obtain plasma and PBMCs. PBMCs were isolated through Ficoll-Paque Plus (Cytiva, cat. 17-1440-03) density gradient centrifugation. Monocytes were enriched by positive selection using CD14-coated microbeads (Miltenyi Biotec). CD14-depleted fractions were stained on ice for 15–20 min to isolate CD19^+^IgG^+^ B cells and total memory CD4^+^ cells (excluding CD45RA^+^, CD25^bright^ and CD8^+^) by sorting to over 98% purity on a FACSAria III (BD Biosciences). The following fluorochrome-labeled mouse mAbs were used for staining: CD4-PE Texas Red (clone S3.5; cat. MHCD0417), CD45RA-Qdot 655 (clone MEM-56; cat. Q10069) from Thermo Fisher, CD8-FITC (clone B9.11; cat. A07756) from Beckman Coulter, CD19-PE-Cy7 (clone SJ25C1; cat. 341113), CD25-PE (clone M-A251; cat. 555432) from BD Biosciences, CCR7-BV421 (clone G043H7; cat. 353208) from BioLegend, Alexa Fluor 647-conjugated goat anti-human IgG (cat. 109-606-170) from Jackson ImmunoResearch. T cells from fresh liver biopsies with confirmed cellular infiltrate at histological examination were polyclonally expanded with 1 μg/mL phytohemagglutinin-L (PHA) (Remel) in the presence of allogenic irradiated (45 Gray) PBMCs (feeder cells) (1 × 10^5^ per well) and IL-2 (500 IU/mL) in a 96-well plate format. On day 15, expanded T cells were labelled with CD3-PE, CD4-PE Texas Red, CD8-APC, CD19-FITC, and CD3^+^CD4^+^CD8^–^CD19^–^ and CD3^+^CD4^–^CD8^+^CD19^–^ T cells were sorted to over 98% purity on a FACSAria III (BD Biosciences), frozen in freezing medium and stored at –150°C.

### Detection of SepSecS-reactive antibodies using SepSecS transfectants.

A synthetic gene expressing full-length SepSecS (501 aa; 55.73 kDa) was produced by Genscript and subcloned into a pcDNA3.1(+)-P2A-eGFP vector. Polyethylenimine (PEI) was used for transient transfection of EXPI293F cells (Gibco), and cells were permeabilized and fixed 60 hours after transfection using BD Cytofix/Cytoperm (cat. 554714) according to the manufacturer’s instructions. SepSecS transfectants were stained on ice for 15–20 minutes with B cell supernatant, blood plasma, or recombinantly produced antibodies. Antibodies bound to SepSecS were detected using a secondary mAb (Alexa Fluor 647-conjugated goat anti-human IgG). Transfected and nontransfected Expi293F cells were distinguished by their eGFP expression.

### Profiling of anti-SepSecS memory B cell repertoire.

3 × 10^4^ PBMCs (AIH11: 2 × 10^4^) were plated in replicate 192 (AIH11: 96) U-bottom wells (Corning, cat. 3799) and stimulated with 500 U/mL IL-2 (produced in house from transfected J558L cells) and 2.5 μg/mL of the Toll-like receptor 7 and 8 agonist R848 (Invitrogen, cat. tlrl-r848-5). After 12 days, the supernatant of each well was screened for the presence of secreted SepSecS-specific IgG using SepSecS-transfectants. After culturing for 12 days, supernatants were screened by flow cytometry using SepSecS-transfectants. Mean APC-A (SepSecS^+^) / mean APC-A (SepSecS^–^) ratio was calculated to exclude unspecific binding by polyreactive antibodies, and ratios above 1.1 were considered positive. Frequency of SepSecS-specific memory B cells was calculated according to the Poisson distribution.

### SepSecS production and purification.

A synthetic gene expressing full-length SepSecS with a C-terminal (G_4_S)_2_ linker and 3× flag tag (SepSecS-GGGGSGGGGSDYKDHDGDYKDHDIDYKDDDDK; 533 aa; 59.07 kDa) was produced by Genscript and subcloned into a pcDNA3.1(+) vector. Polyethylenimine (PEI) was used for transient transfection of EXPI293F cells (Gibco), and cells were lysed by rotating at 4°C for 2 hours in lysis buffer (50 mM Tris HCl, pH 7.4, 250 mM NaCl, 1 mM EDTA, 0.5% Triton X-100). Lysate was clarified by centrifugation at 16,000*g*, and supernatant was incubated for 8 hours with anti-FLAG magnetic beads (Merck, cat. M8823). Beads were washed 3× with TBS and SepSecS was eluted by rotating at 4°C for 5 hours in TBS containing 100 ng/μL 3× FLAG Peptide (Merck, cat. F4799). Buffer was exchanged to storage buffer (25 mM Tris, pH8.0, 150 mM NaCl, 10% glycerol) and peptides were removed by dialysis. Purified protein was stored in aliquots at –20°C. Identity and purity of SepSecS were tested by Coomassie electrophoresis gel staining and Western blot using rabbit anti-SepSecS primary and HRP-conjugated donkey anti-rabbit secondary antibodies (Abcam, cat. ab236956 and Jackson ImmunoResearch, cat. 711-035-152). If not stated otherwise, “SepSecS” always refers to this, in human cells, recombinantly produced SepSecS.

### ELISA.

MaxiSorp 96-well high protein binding plates (Nunc) were coated for 16 hours at 4°C with 5 μg/mL SepSecS. Plates were subsequently washed and blocked with 1% BSA in PBS and incubated with titrated mAbs, B cell supernatants, or plasma. Plates were then washed and incubated with 1:500 diluted alkaline phosphatase-conjugated goat anti-human IgG (SouthernBiotech, cat. 2040-04). Substrate (para-nitrophenyl phosphate (p-NPP), Sigma-Aldrich), was added and plates were read at 405 nm to determine optical density (OD). For competition ELISA, primary mAbs were added in excess to block mAb-specific epitopes completely. Biotinylated mAbs were added as secondary mAbs and binding was detected using streptavidin-HRP (Cytiva, cat. RPN1231-100UL). For IgG quantification, MaxiSorp 96-well high protein binding plates (Nunc) were coated for 16 hours at 4°C with 10 μg/mL goat anti-human IgG (SouthernBiotech, cat. 2040-01). Plates were subsequently washed and blocked with 1% BSA in PBS and incubated with titrated mAbs or B cell supernatants using Certified Reference Material 470 (Sigma-Aldrich, cat. ERMs-DA470) as standard. Plates were then washed and incubated with 1:500 diluted alkaline phosphatase-conjugated goat anti-human IgG (SouthernBiotech, cat. 2040-04). Substrate (para-nitrophenyl phosphate (p-NPP), Sigma-Aldrich), was added and plates were read at 405 nm to determine optical density (OD). Commercial ELISA to detect anti-SepSecS serum antibodies (Euroimmun, cat. EA 1302-9601 G) was performed according to manufacturer’s instructions.

### Immortalization of B cells using EBV.

CD19^+^IgG^+^ memory B cells were immortalized using EBV and plated in single-cell cultures in the presence of CpG-2006 (2.5 μg/mL) and irradiated PBMCs, as previously described (14). Two weeks after immortalization, culture supernatants were screened for SepSecS-binding antibodies by flow cytometry using SepSecS transfectants. B cell cultures that tested positive were tested a second time to acquire a dilution curve, and IgG concentration in the supernatant was assessed by ELISA as described above. Some cultures that tested positive were expanded and/or sequenced.

### BCR and TCR Vβ sequencing.

B cell receptor (BCR) sequencing was performed as described by Tiller et al. (44). Briefly, cDNA was synthesized by reverse transcription PCR (RT-PCR) of total RNA from one or more B cells, using separate VH, VK, and VL-specific RT primers. VH, VK, and VL sequences were amplified by PCR using VH/VK/VL primers (Supplemental Table 6). Sequence amplification was assessed by agarose gel electrophoresis. Sanger sequencing of successfully amplified fragments was performed by Microsynth. Sequencing of rearranged TCR Vβ was performed as described previously (45). cDNA was synthesized by RT-PCR of total RNA from 1 × 10^3^ T cells per reaction. Rearranged TCR Vβ genes were PCR amplified using a forward primer pool targeting Vβ and reverse primer pairing to C1–C2 β-chain constant region (Supplemental Table 6). Sequence amplification was assessed by agarose gel electrophoresis; Sanger sequencing of successfully amplified fragments was performed by Microsynth.

### Deep TCR Vβ sequencing.

1 × 10^6^ PBMCs taken on the same day as the liver biopsy of patient 12 were analyzed by deep sequencing. PBMCs were washed in PBS, and genomic DNA was extracted from the pellet according to manufacturer’s instructions using the QIAamp DNA Micro Kit (Qiagen). DNA quantity and purity were assessed by spectrophotometric analysis. Using the ImmunoSEQ platform of Adaptive Biotechnologies TCR Vβ CDR3 sequences were obtained (http://www.immunoseq.com). First, all CDR3 Vβ fragments are amplified by multiplex PCR, then, using the Illumina HiSeq platform, amplicons were sequenced, resulting in nucleotide and amino acid sequences containing the CDR3 region. Clonotypes were defined as having a unique productively rearranged TRBV nucleotide sequence. Data were processed using ImmunoSEQ Analyzer V.3.0 providing productive frequency of reads (http://www.immunoseq.com).

### BCR/TCR sequence analysis.

BCR and TCR sequence annotation was carried out, alignment to germline sequences was performed, and unmutated common ancestor (UCA) antibody sequences were constructed using the IMGT/V-QUEST database (version 3.4.17) (46). Bayesian analysis for ancestral rearrangement was performed on 10 antibodies of P12, which belong to 4 clonal families using the software Cloanalyst from the Laboratory of Computational Immunology of Boston University (www.bu.edu/computationalimmunology/cloanalyst).

### Recombinant mAb production.

mAbs to be produced recombinantly were selected based on their diverse gene usage and affinity to SepSecS (EC_50_ values between 1 and 20 ng/mL). Vectors encoding antibody heavy and light chains were obtained in 2 different ways. In the case of clone 9 of patient AIH11, PCR amplified variable regions of the heavy and κ chains were cloned into vectors encoding human IgG1 and Igκ, respectively, using 1 Shot competent *E*. *coli* (Thermo Fisher Scientific, cat. C737303). The cloned plasmids were validated by Sanger sequencing (Microsynth). All other plasmids, including all germline-encoding plasmids, were produced by Genscript and subcloned into vectors for expression of human IgG1, Igκ, and Igγ. Antibodies were expressed by transient transfection of EXPI293F cells (Gibco) with antibody heavy and light chains using PEI. Affinity purification was carried out using ÄKTA Pure 25 (Cytiva) operated with UNICORN 6.4. Columns were HiTrap Protein A (Cytiva, cat. GE17-5079-01) for antibody purification and HiPrep 26/10 Desalting (Cytiva, cat. GE17-5087- 01) for buffer exchange to PBS. Desalted antibodies were sterilized by filtration. In some cases, purified mAbs were biotinylated using Pierce Antibody Biotinylation Kit for IP (Thermo Fisher Scientific, cat 15332617), according to manufacturer’s instructions.

### Ex vivo T cell stimulation and isolation of autoreactive T cell clones.

Memory CD4^+^ T cells isolated from PBMCs or from expanded T cells from liver biopsies were labelled with CFSE and cultured at a ratio of 2:1 with irradiated autologous monocytes untreated or pulsed for 2 hours with 1 μg/mL of a pool of overlapping 20-mer peptides spanning the whole sequence of SepSecS (Biosynth; numbering according to UniProtKB sequence Q9HD40). On day 7, cells were stained with antibodies against CD25-PE and ICOS-Pacific Blue (BioLegend). To isolate SepSecS-reactive CD4^+^ T cell clones, CFSE^lo^CD25^+^ICOS^+^ T cells were sorted and plated with limiting dilution (0.5 cells/well) in 384-well plates with 1 μg/mL PHA (Remel) in the presence of allogenic irradiated (45 Gray) PBMCs (feeder cells, 25,000 per well) and IL-2 (500 U/mL). T cell clones’ specificity was tested by stimulation with irradiated autologous B cells untreated or pulsed for 2 hours with 1 μg/mL of a pool of overlapping 20-mer peptides spanning the whole sequence of SepSecS. T cell proliferation was measured at day 3 after a 16 hour incubation with 1 μCi/mL [methyl-^3^H]thymidine (Perkin Elmer). Epitope mapping experiments were performed by stimulation of autoreactive T cell clones with irradiated autologous B cells after 2 hour pulsing with 20-mer overlapping peptide pools of 10 peptides each (1 μg/mL). T cell clone proliferation was measured at day 3 after 16 hours’ incubation with 1 μCi/mL [methyl-^3^H]thymidine (Perkin Elmer) by counting the incorporated radioactivity as counts per minute (cpm).

### Antigen presentation by B cells to T cells.

1.4 × 10^4^ SepSecS-specific CD4^+^ T cells of patient AIH12 were cultured with 7 × 10^3^ irradiated (45 Gy), autologous, EBV-transformed B cells, which were pulsed with defined concentrations of SepSecS. Proliferation was measured on day 3 after incubation for 16 hours with 1 μCi/mL [methyl-^3^H]-thymidine (Perkin Elmer) by counting the incorporated radioactivity as cpm.

### Luminex.

Cytokine concentrations in the 48 hour culture supernatants of antigen-stimulated CD4^+^ T cell cultures were assessed by Luminex bead-based assay (Thermo Fisher Scientific) according to the manufacturer’s instructions.

### Statistics.

Statistical analysis was performed with Prism Graph Pad 9.4.1. Continuous variables are expressed as median, interquartile range (IQR), and range; categorical variables are expressed as numbers and percentages. The Fisher exact test was used to compare categorical data between 2 groups. The Mann-Whitney U test or the unpaired 2-tailed *t* test was used to compare quantitative data between 2 groups, and the Spearman Partial correlation was used in Figure 4B considering age as a covariable. *P* values below 0.05 (2-tailed) were considered significant in all analyses. EC_50_ (ng/mL) values were calculated by nonlinear regression curve fit (4PL with least squares regression) using GraphPad Prism 9.4.1 software.

### Study approvals.

Written informed consent was obtained from each patient or guardian before sample and data collection. The study protocol conforms to the ethical guidelines of the 1975 declaration of Helsinki and was approved by the local Ethics Committee (Comitato etico Canton Ticino, no. 2019-01766 / CE 3520). The project was also approved by the Scientific Committee of the Swiss AIH Cohort Study. Human primary cell protocols were approved by the Federal Office of Public Health (no. A000197/2 to FS.).

### Data availability.

Values for all data points in graphs are reported in the Supporting Data Values file. TCR Vβ sequences from samples in Figure 5C are shared through the immuneACCESS data portal (https://www.immunoseq.com/immuneaccess/). Data are available at https://clients.adaptivebiotech.com/pub/kramer-2024-jci; http://doi.org/10.21417/MK2024JCI

## Author contributions

MK, BTBP, FM, SFL, JSL, and DJ performed experiments, and, with assistance from SJ and BMF, acquired data and prepared the figures with assistance from AC and FS. BTBP, CS, and MFS recruited participants, performed clinical evaluation, and collected biological samples. MK, BTBP, and FS analyzed the data and wrote the original draft with contributions from GMV, DV, AL, and AC. All authors provided input and critical revision of the manuscript.

## Supplementary Material

Supplemental data

Supporting data values

## Version 1 01/16/2025

Electronic publication
