# Supplementary material for: Clonal analysis of SepSecS-specific B and T cells in autoimmune hepatitis
Source: J Clin Invest. 2025 Jan 16;135(2):e183776. doi: 10.1172/JCI183776 (PMC11735102; doi:10.1172/JCI183776)
Supplement: Supplemental data [file jci-135-183776-s098.pdf]

Supplemental Information for:

Clonal analysis of SepSecS-specific B and T cells in autoimmune hepatitis

Michael Kramer[1], Federico Mele[1], Sandra Jovic[1], Blanca Maria Fernandez[1], David Jarrossay[1], Jun Siong Low[2], Christiane Sokollik[3], Magdalena Filipowicz Sinnreich[4,5], Sylvie Ferrari-Lacraz[6], Giorgina Mieli-Vergani[7], Diego Vergani[7], Antonio Lanzavecchia[8], Antonino Cassotta[1], Benedetta Terziroli Beretta-Piccoli[1,9,10], Federica Sallusto[1,2,9]

[1]Institute for Research in Biomedicine (IRB), Bellinzona, Switzerland

[2]Institute of Microbiology, ETH Zurich, Zurich, Switzerland

[3]Division of Paediatric Gastroenterology, Hepatology and Nutrition, Department of Paediatrics, Inselspital, Bern University Hospital, University of Bern, Switzerland

[4]Department of Gastroenterology and Hepatology, Basel University Medical Clinic, Cantonal Hospital Baselland, Liestal, Switzerland

[5]Department of Biomedicine, University of Basel, Switzerland

[6]Transplant Immunology Unit & National Laboratory of Immunogenetics, Division of Nephrology, Department of Diagnostic, University Hospital Geneva, Geneva, Switzerland

[7]MowatLabs, Faculty of Life Sciences & Medicine, King's College London, King's College Hospital, London, UK

[8]National Institute of Molecular Genetics, Milano, Italy

[9]Faculty of Biomedical Sciences, Università della Svizzera italiana, Lugano, Switzerland

[10]Epatocentro Ticino, Lugano, Switzerland

Authorship note: BTB-P and FS contributed equally to this work

This PDF file includes:

- Supplemental Figures 1-5
- Supplemental Tables 1-6

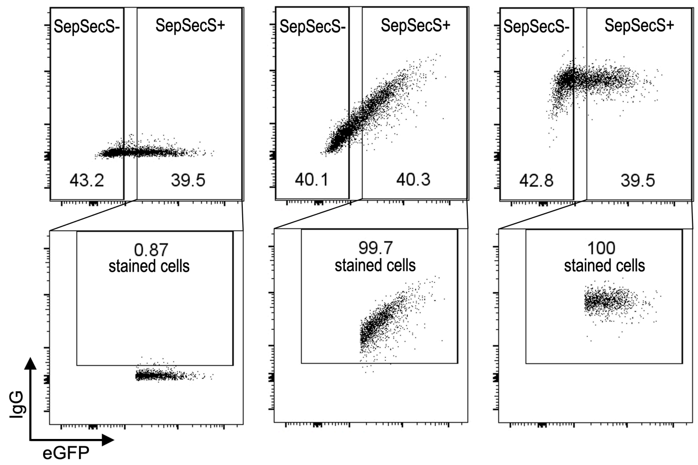

Supplemental Figure 1. Detection of SepSecS-reactive IgG in human sera using eGFP- and SepSecS-transfected EXPI-293 cells. Bound IgG was detected using Alexa Fluor 647-labeled anti-IgG antibodies. The 3 FACS plots are representative of sera with no SepSecS-binding antibodies (left), with SepSecS-specific binding antibodies (center, higher binding to transfected than non-transfected cells), and with unspecific-binding antibodies (right, equal binding to transfected and non-transfected cells).

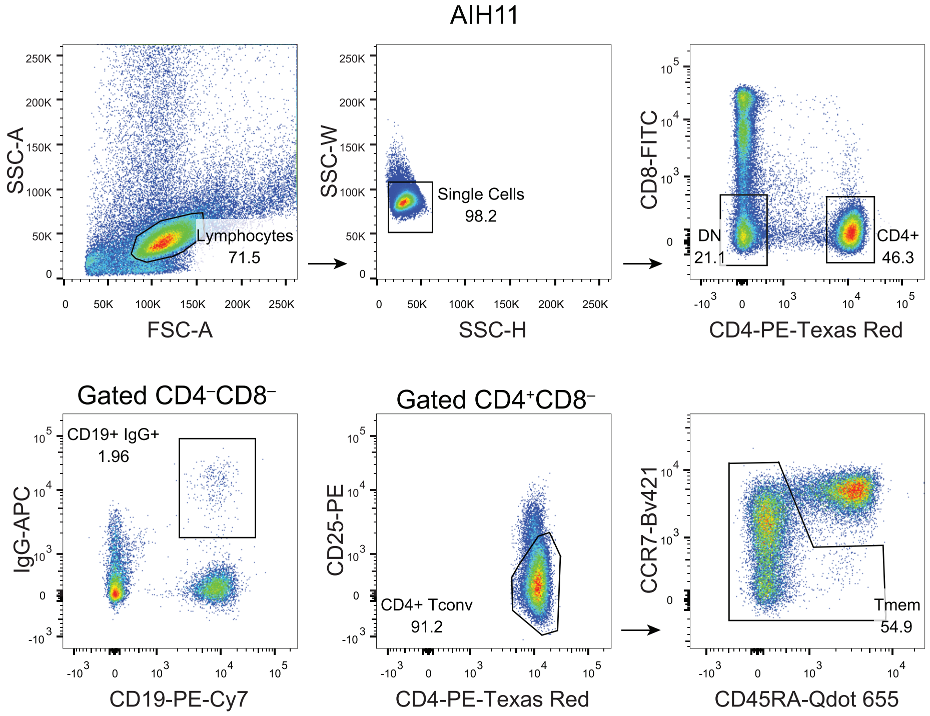

Supplemental Figure 2. Sorting of B cells and T cells from PBMCs. Sorting strategy to isolate IgG^{+} B cells and total memory CD4^{+} T cells from PBMCs. Representative dot plots from the sorting of PBMC sample from patient AIH11. Numbers represent percentages of gated populations.

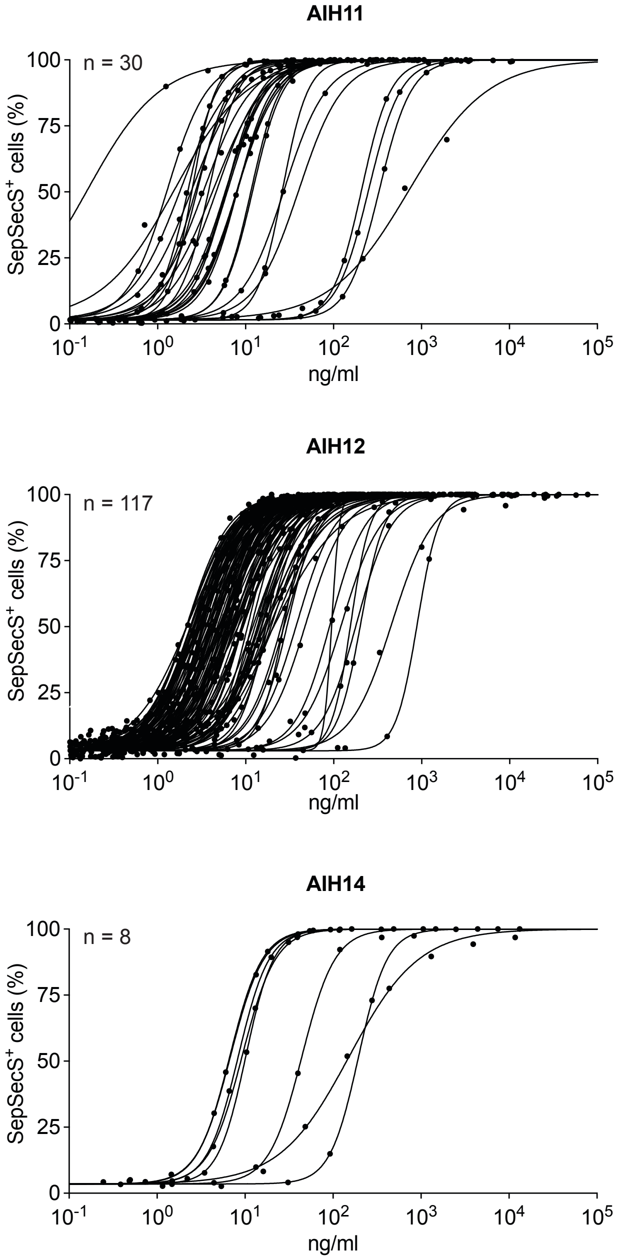

Supplemental Figure 3. Binding of SepSecS-reactive monoclonal antibodies (mAbs) from AIH patients. Binding of antibodies in supernatants of EBV-transformed B cell clones from patients AIH11, AIH12 and AIH14 to SepSecS-transfectants as measured with flow-cytometry. The number of mAbs tested is also indicated.

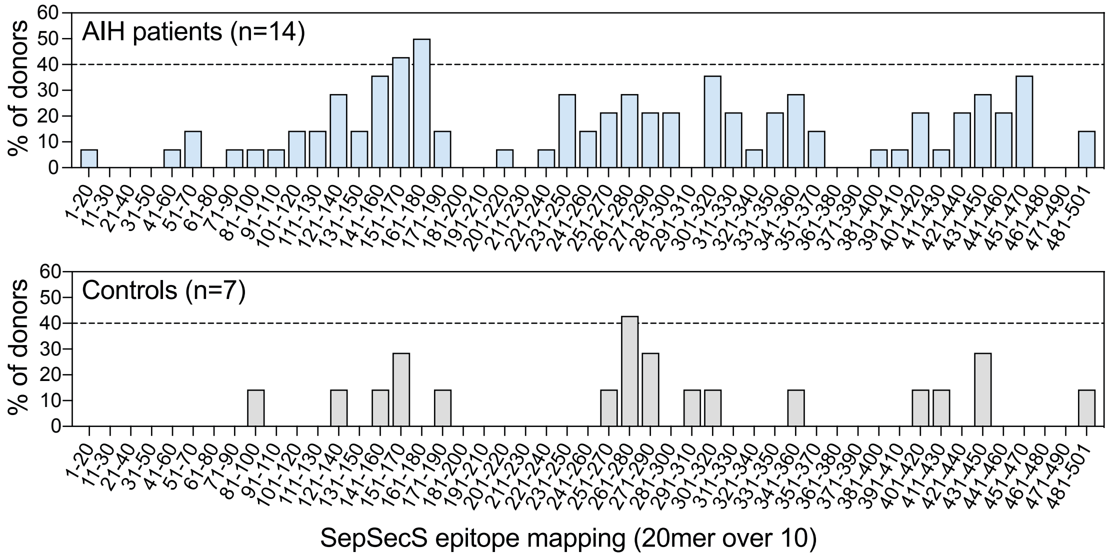

Supplemental Figure 4. Percentage of individuals carrying T cell clones specific for different 20-mer segments of the SepSecS protein. The upper barplot represents AIH patients (n=14), while the lower barplot shows the control group (n=7).

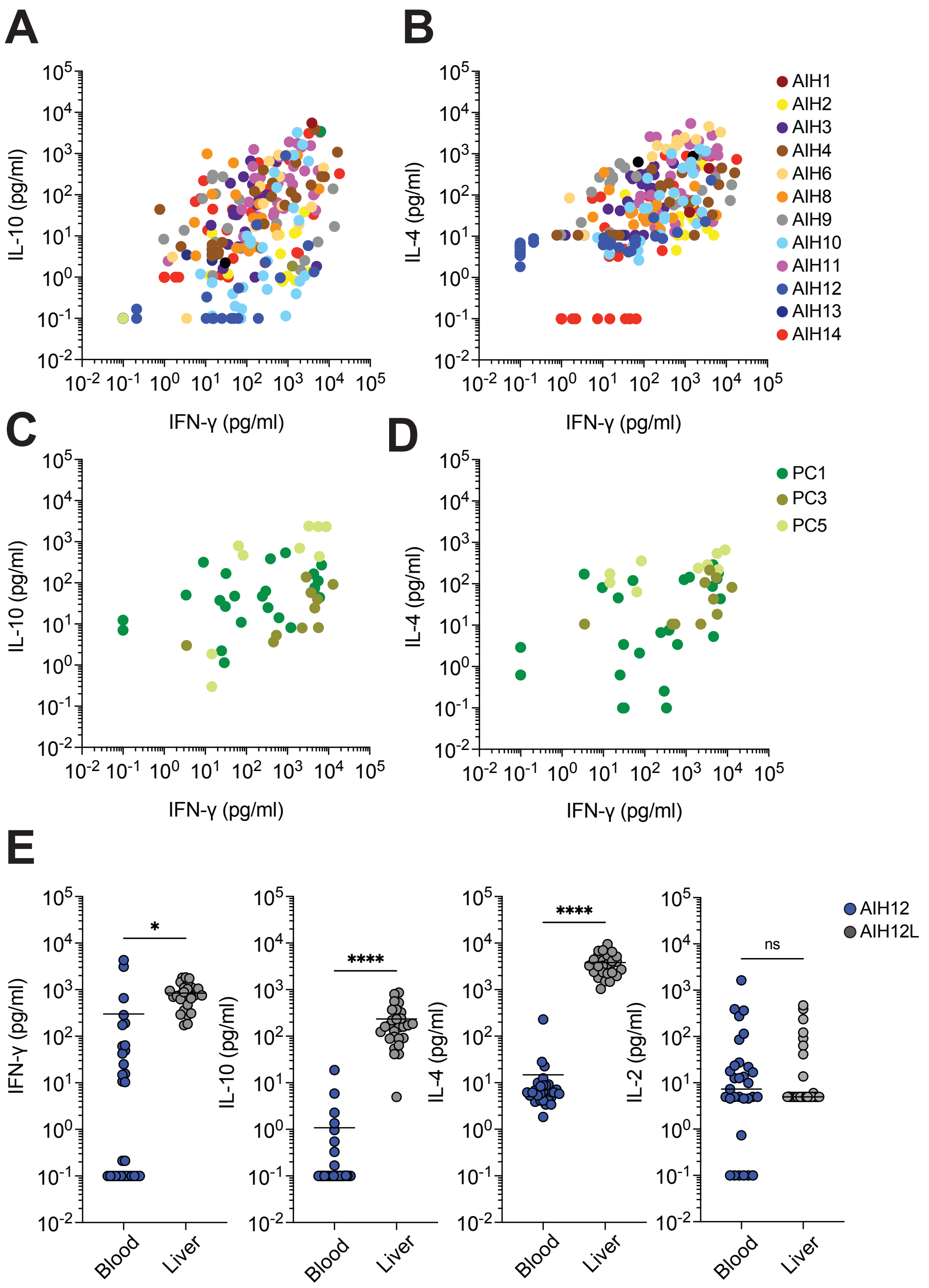

Supplemental Figure 5. (**A-D**) Cytokine production of SepSecS-specific CD4$^+$ T cell clones from AIH patients (A, B) and pathological controls (C, D). (**E**) Cytokine production of SepSecS-specific CD4$^+$ T cell clones isolated from blood (already displayed in panels A, B) or liver biopsy (L) from patient AIH12. Cytokine concentration in the 48 h culture supernatants (obtained from epitope mapping experiments) was measured by Luminex assay. *p-value=0.01, **** p-value<0.0001, n.s. not significant, determined by unpaired t test.

Supplemental Table 1. Clinical features of the study population and of pathological controls

ID	Gender	Age (y)	Disease	Anti-SLA[A]	IAIHG score	Disease duration (y)	At time of blood donation: Normal transa-minases	At time of blood donation: IS treatment	HLA DRB1	
**AIH1**	M	13	AIH-2	–	7	8	Yes	Aza	04:01	07:01
**AIH2**	F	69	AIH-1	–	6	1	Yes	PDN + MMF	03:01	13:01
**AIH3**	M	73	AIH-1	–	7	12	Yes	PDN + Aza	07:01	14:01
**AIH4**	F	73	AIH-1	+	8	10	Yes	PDN + Aza	04:04	13:01
**AIH5**	F	23	AIH-2	–	8	9	Yes	PDN + Aza	12:01	15:01
**AIH6**	F	72	AIH-1	–	7	6	Yes	PDN	04:01	08:01
**AIH7**	F	50	AIH-1	–	8	5	No	PDN + MMF	03:01	13:02
**AIH8**	F	27	AIH-1	–	7	4	No	PDN + MMF	11:01	15:01
**AIH9**	F	68	AIH-1	–	8	3	Yes	Aza	11:01	16:01
**AIH10**	F	67	AIH-1	–	6	8	Yes	PDN + MMF	08:01	15:02
**AIH11**	F	54	AIH-1	+	8	10	Yes	PDN + MMF	03:01	03:01
**AIH12**	F	48	AIH-1	+	8	28	No	PDN, MMF, Tac	03:01	03:01
**AIH13**	F	72	AIH-1	–	6	5	Yes	PDN + MMF	04:01	15:01
**AIH14**	F	25	AIH-1	+	6	3	Yes	PDN	13:01	15:01
**AIH15**	F	59	AIH-1	–	6	2	No	PDN + Aza	04:01	16:01
**AIH16**	F	53	AIH-1	–	4	0	No	None	03:01	08:01
**AIH17**	F	31	AIH-2	+	8	26	No	PDN + Aza	07:10	07:10
**AIH18**	F	27	AIH-1	–	8	11	Yes	PDN + Aza	03:01	03:01
**AIH19**	F	32	AIH-1	+	8	0	No	None	04:05	13:01
**AIH20**	M	18	AIH-1	+	7	0	No	None	01:02	13:01
**Total**	**F:M 17:3**	**Median 51.5**	**AIH-1: AIH-2 17:3**	**7 (35%)**	**Median 7**	**Median 5.5**	**No=8 Yes=12**	**IS=17 None=3**		
**PC1**	F	65	DILI	No	n.d.	7	Yes	None	04:05	13:02
**PC2**	F	47	PBC	No	n.d.	2	Yes	None	07:01	07:01
**PC3**	F	39	PBC	No	n.d.	10	Yes	None	07:01	13:01
**PC4**	F	26	PBC	No	n.d.	2	Yes	None	10:01	13:01
**PC5**	M	75	PSC	No	n.d.	10	Yes	None	03:01	16:02
**PC6**	F	77	PBC	No	n.d.	3	Yes	None	n.d.	n.d.
**PC7**	F	66	PBC	No	n.d.	5	Yes	None	08:01	11:01
**PC8**	F	54	PBC	No	n.d.	12	No	None	04:01	04:07
**PC9**	M	71	PBC	No	n.d.	0	No	None	03:01	08:01
**Total**	**F:M 7:2**	**Median 65**	**PBC=7**			**Median 5**	**No=2 Yes=7**	**None=9**		

[A]Tested at local hospitals. ID, patient identification code. AIH, autoimmune hepatitis patient. PC, pathological control. F, female. M, male. DILI, Drug-induced autoimmune liver injury. PBC, primary biliary cholangitis. PSC, primary sclerosing cholangitis. Aza, azathioprine. PDN, prednisone. Tac, Tacrolimus. MMF, mycophenolate mofetil. Anti-SLA, anti-soluble liver antigen antibody. IAIHG, international AIH group. IS, immunosuppression. HLA, human leukocyte antigens. n.d., not determined. AIH12 had undergone liver transplantation for severe autoimmune hepatitis, and experienced post-transplant recurrence at the time of blood collection.

Supplemental Table 2. Anti-SLA antibodies measured in AIH patients and controls

Donor	Anti-SLA	Commercial ELISA		In house assay ELISA		In house assay FACS		Frequency SepSecS-IgG+ B cells In 10^6 PBMC
		OD450 1:800	Units	OD405 1:800	EDF_{50}	MFI 1:800	EDF_{50}	
**AIH1**	–	0.06	5	0.14	<50	620	<50	0.2
**AIH2**	–	0.05	<2	0.12	<50	1001	<50	1.1
**AIH4**	+	0.50	155	4.19	8973	22145	29114	23.8
**AIH5**	–	0.05	<2	0.12	<50	760	<50	0.2
**AIH6**	–	0.05	<2	0.15	<50	1100	<50	0.2
**AIH7**	–	0.05	<2	0.11	<50	931	<50	0.2
**AIH9**	–	0.06	<2	0.20	<50	1055	<50	0.5
**AIH11**	+	0.72	293	4.22	46199	63367	67358	16.5
**AIH12**	+	1.03	496	4.28	50005	67959	88038	129.0
**AIH13**	–	0.05	<2	0.27	53	951	<50	0.0
**AIH14**	+	0.08	9	4.18	9748	26087	16327	42.3
**AIH18**	–	0.05	<2	4.15	6748	14623	16944	43.5
**HC3**	n.d.	0.05	<2	0.11	<50	724	<50	0.3
**HC12**	n.d.	0.05	<2	0.15	<50	998	<50	0
**HC13**	n.d.	0.05	<2	0.12	<50	527	<50	0
**HC14**	n.d.	0.07	<2	0.11	<50	342	<50	0
**HC15**	n.d.	0.07	<2	0.13	<50	828	<50	3.1

Supplemental Table 3. V(D)J gene usage and EC_{50} values of SepSecS-binding mAbs

ID	B cell clone	Isotype		Heavy chain VDJ genes (% identity to germline)					Light chain VJ genes (% identity to germline)				EC_{50} (ng/ml)
**AIH11**	**9**	IgG1	κ	VH3-20	89.2	D3-22	JH4	85.4	VK1-39	93.9	JK1	91.7	4.0
	**1**	IgG1	κ	VH1-69	94.4	D3-22	JH6	88.7	VK1-5	95.3	JK1	89.5	4.3
	**2**	IgG1	κ	VH1-3	92.7	D2-2	JH6	82.3	VK1-39	95.0	JK4	100	2.4
	**4**	IgG3	λ	VH3-49	95.2	D3-10	JH6	87.1	VL1-51	97.5	JL3	97.1	7.1
	**5**	IgG2	λ	VH4-4	95.5	D2-8	JH4	91.7	VL2-11	94.8	JL3	91.9	2.2
	**6**	IgG1	κ	VH4-31	91.1	D6-13	JH4	83.3	VK3-20	94.3	JK5	94.3	28.0
	**7**	IgG2	λ	VH5-10-1	96.2	D6-6	JH3	94.0	VL2-23	94.8	JL3	100	2.2
	**8-1**	IgG1	κ	VH3-33	86.8	D6-19	JH4	95.8	VK1-5	93.6	JK1	100	3.5
	**8-2**	IgG1	κ	VH3-33	87.5	D6-19	JH4	95.8	VK1-5	93.9	JK1	100	2.6
	**9**	IgG1	κ	VH3-30	88.9	D6-19	JH4	93.8	VK1-5	94.6	JK1	94.7	6.1
	**10**	IgG1	κ	VH4-59	96.8	D3-22	JH2	92.5	VK3-15	97.5	JK2	94.9	4.0
	**12**	IgG1	κ	VH4-34	95.4	D4-23	JH4	91.7	VK4-1	97.3	JK1	97.4	5.2
	**13**	IgG1	λ	VH3-30	95.5	D3-3	JH6	77.4	VL1-44	96.1	JL2	89.2	3.2
	**14**	IgG1	κ	VH1-46	86.5	D3-22	JH3	98.0	VK1-39	90.0	JK2	91.4	5.1
	**15**	IgG1	κ	VH1-46	86.5	D6-19	JH3	98.0	VK1-39	90.0	JK4	80.6	3.7
	**16**	IgG1	κ	VH3-23	89.9	D3-22	JH4	97.9	VK3-15	97.1	JK3	97.1	3.1
	**18**	IgG1	λ	VH3-20	89.6	D1-26	JH4	87.5	VL3-21	95.7	JL3	94.7	8.0
	**19**	IgG1	κ	VH3-20	92.0	D3-22	JH4	97.9	VK1-5	93.9	JK2	87.2	3.4
	**20**	IgG1	κ	VH3-7	90.6	D2-2	JH6	80.7	VK4-1	95.0	JK1	100	4.4
	**21**	IgG1	κ	VH3-23	89.6	D1-14	JH6	85.5	VK2-30	96.3	JK2	100	4.3
	**22**	IgG1	κ	VH3-20	94.8	D3-10	JH5	90.2	VK3D-20	96.5	JK4	100	13.9
	**23**	IgG2	κ	VH3-15	93.5	D5-18	JH4	83.3	VK1-5	95.3	JK1	97.4	2.2
	**24**	IgG1	λ	VH3-20	92.0	D3-10	JH5	98.0	VL1-40	94.1	JL3	83.3	15.4
	**25**	IgG1	λ	VH3-23	89.9	D3-9	JH4	87.5	VL3-10	96.1	JL3	94.7	460.9
	**26**	IgG1	κ	VH3-23	95.5	D6-13	JH4	85.4	VK1-5	97.5	JK1	94.7	9.4
	**27**	IgG1	κ	VH3-23	86.7	D3-16	JH4	87.5	VK1-39	93.2	JK4	100	4.0
	**28**	IgG2	κ	VH3-20	92.4	D5-18	JH4	91.7	VK2-30	95.2	JK2	92.3	15.7
	**29**	IgG1	λ	VH4-34	90.9	D2-2	JH6	83.9	VL3-1	93.2	JL2	94.7	17.4
	**30**	IgG1	κ	VH3-23	83.3	D6-19	JH1	76.9	VK1-6	94.3	JK1	97.2	13.1
**AIH12**	**31**	IgG1	κ	VH3-7	89.6	D2-15	JH4	87.5	VK3-15	96.1	JK1	97.4	12.4
	**32-1**	IgG2	λ	VH3-23	86.3	D3-10	JH3	62.0	VL1-51	91.6	JL3	81.6	5.4
	**32-2**	IgG2	λ	VH3-23	86.7	D3-10	JH3	62.0	VL1-51	88.1	JL3	83.8	6.4
	**32-3**	IgG2	λ	VH3-23	85.6	D3-10	JH3	62.0	VL1-51	90.9	JL3	76.3	0.4
	**33-1**	IgG1	κ	VH3-23	89.9	D3-3	JH3	86.0	VK3-15	95.0	JK1	85.7	6.0
	**33-2**	IgG1	κ	VH3-23	92.0	D3-3	JH3	86.0	VK3-15	95.3	JK1	94.3	3.1
	**33-3**	IgG1	κ	VH3-23	91.7	D3-3	JH3	84.0	VK3-15	94.6	JK1	91.4	2.8
	**34**	IgG1	κ	VH4-34	89.1	D5-18	JH4	91.7	VK3-11	96.4	JK4	100	2.1
	**35**	IgG1	n.d.	VH4-59	93.7	D3-16	JH5	84.3	n.d.	n.d.	n.d.	n.d.	3.2
	**36**	IgG1	κ	VH3-66	88.4	D3-10	JH4	87.5	VK1-39	94.3	JK4	100	5.3
	**37-1**	IgG1	κ	VH1-69	87.2	D5-18	JH4	89.6	VK1-33	93.6	JK5	97.4	2.7
	**37-2**	IgG1	κ	VH1-69	87.9	D5-18	JH4	89.6	VK1-33	92.8	JK5	97.4	4.0
	**38**	IgG1	κ	VH1-69	93.1	D5-18	JH4	83.3	VK1-33	96.1	JK5	100	2.2
	**39**	IgG1	n.d.	VH1-3	92.6	D6-13	JH3	94.0	n.d.	n.d.	n.d.	n.d.	182.9
	**41**	IgG1	κ	VH4-39	91.1	D1-26	JH4	91.7	VK1-5	93.9	JK1	100	9.3
	**42**	IgG1	λ	VH2-26	97.6	D3-9	JH6	95.2	VL3-1	95.7	JL1	94.7	12.4
	**43**	IgG1	κ	VH4-31	91.4	D3-10	JH4	70.5	VK1-5	95.3	JK1	89.5	15.7
	**44**	IgG1	λ	VH4-59	89.1	D3-10	JH5	86.3	VL2-18	96.5	JL3	86.8	2.7
	**45**	IgG2	κ	VH3-11	92.0	D3-10	JH6	77.4	VK1-9	97.1	JK3	94.7	7.7
	**46**	IgG1	λ	VH1-24	91.0	D5-12	JH5	84.3	VL2-18	91.3	JL3	86.8	5.9
	**47**	IgG1	κ	VH3-23	93.4	D6-19	JH4	95.8	VK3-15	98.2	JK1	100	3.1
	**49**	IgG1	κ	VH3-20	92.4	D2-2	JH6	90.3	VK1-16	96.8	JK4	100	17.7
	**50**	IgG1	λ	VH4-31	92.1	D3-10	JH4	85.4	VL7-46	95.5	JL2	100	3.1
	**51**	IgG1	κ	VH3-20	89.1	D6-19	JH6	87.1	VK1-5	93.9	JK1	88.9	0.5
	**53**	IgG1	κ	VH4-61	97.3	D3-9	JH4	85.4	VK1-16	97.5	JK4	94.7	3.5
	**54**	IgG1	λ	VH4-34	91.6	D5-24	JH6	83.9	VL2-14	94.4	JL1	85.3	5.7
	**55**	IgG1	κ	VH4-34	90.9	D3-10	JH6	79.0	VK1-9	96.1	JK1	97.3	2.9
	**56**	IgG1	κ	VH4-4	97.6	D3-9	JH5	86.3	VK1-33	98.9	JK4	100	16.4
	**nb**	IgG1	n.d.	VH1-3	97.9	D3-22	JH3	96.0	n.d.	n.d.	n.d.	n.d.	n.d.
	**7**	IgG1	λ	VH5-10-1	90.3	D3-16	JH3	86.0	VL3-21	89.6	JL1	86.8	8.1
	**8**	IgG1	λ	VH1-3	86.8	D5-24	JH1	90.2	VL3-21	93.2	JL3	100	4.4
	**9**	IgG1	κ	VH3-23	78.1	D6-19	JH4	83.3	VK1-5	95.0	JK1	89.5	10.2
**AIH14**	**13**	IgG1	λ	VH4-39	92.1	D3-10	JH5	98.0	VL3-21	97.1	JL1	92.1	195.1
	**14**	IgG1	λ	VH2-70	93.1	D5-24	JH4	97.9	VL3-21	94.3	JL1	94.6	152.4
	**19**	IgG1	κ	VH3-23	85.4	D5-12	JH4	81.3	VK3-11	90.6	JK3	94.3	3.2
	**20**	IgG1	λ	VH4-59	82.8	D2-8	JH5	78.4	VL1-36	86.4	JL1	84.2	8.6
	**21**	IgG1	κ	VH3-33	93.8	D5-24	JH4	91.7	VK1-5	91.4	JK2	86.8	17.0

EC_{50} values based on SepSecS-transfected EXPI293 cells; n.d., not determined.

Supplemental Table 4. Summary of CD4^{+} T cell clones obtained from AIH patients and controls

ID	Ex vivo T cell proliferation CFSE^{low}ICOS^{+}CD25^{+} (%)	Isolated T cell clones n	SepSecS-specific T cell clones n	%
**AIH1**	0.32	10	1	10
**AIH2**	0.27	53	15	28
**AIH3**	11.16	120	117	97
**AIH4**	1.15	200	147	73
**AIH5**	7.40	120	86	72
**AIH6**	0.42	60	26	43
**AIH7**	2.10	203	86	42
**AIH8**	1.33	96	13	13
**AIH9**	1.81	136	55	40
**AIH10**	0.06	30	27	90
**AIH11**	0.90	31	31	100
**AIH12**	22.30	180	168	93
**AIH13**	1.68	32	6	19
**AIH14**	0.54	93	52	56
**AIH15**	0.1	n.d.	–	–
**AIH16**	0.1	n.d.	–	–
**AIH17**	0.1	n.d.	–	–
**AIH18**	0.1	n.d.	–	–
**AIH19**	0.1	n.d.	–	–
**AIH20**	0.1	n.d.	–	–
**AIH12 LB**	11.7*	130	87	66.9
**AIH16 LB**	11.9*	41	0	0
**PC1**	0.47	96	13	13
**PC2**	0.77	96	24	25
**PC3**	0.09	39	10	26
**PC4**	0.36	120	32	27
**PC5**	0.78	96	64	67
**PC6**	0.05	n.d.	–	–
**PC7**	0.02	n.d.	–	–
**PC8**	0.06	n.d.	–	–
**PC9**	0.1	n.d.	–	–
**HC1**	0.20	96	15	16
**HC2**	0.03	192	73	38
**HC3**	0.52	10	1	10
**HC4**	0.1	n.d.	–	–
**HC5**	2.57	14	2	14
**HC6**	0.02	n.d.	–	–
**HC7**	0.15	n.d.	–	–
**HC8**	0.06	n.d.	–	–
**HC9**	0.1	n.d.	–	–
**HC10**	3.29	n.d.	–	–
**HC11**	0.1	n.d.	–	–
**HC16**	0.12	n.d.	–	–

AIH, autoimmune hepatitis patient. PC, pathological control. HC, healthy control. n.d., not done.
*Proliferation of liver biopsy-derived T cell lines

Supplemental Table 5. Cytokines produced by SepSecS-specific $CD4^+$ T cell clones from AIH patients and controls

ID	Clone n.	IFN-γ (pg/ml)	IL-10 (pg/ml)	IL-4 (pg/ml)
AIH1	1	3809.8	5512.5	446.4
	2	1287.1	1.9	38.1
AIH2	1	12.9	1.1	10.6
	2	663.2	3.7	10.6
	3	1521.9	0.8	65.2
	4	3441.2	1.4	15.3
	5	1614.2	12.5	24.4
	6	2540.4	38.4	533.7
	7	14.4	11.6	11.6
	8	700.4	1.0	20.2
	9	984.4	1.1	10.6
	10	35.1	1.2	102.4
	11	618.4	10.7	4.6
	12	1433.4	11.7	29.6
	13	738.5	0.8	29.9
	14	4977.7	1.8	10.6
AIH3	1	176.7	57.0	307.7
	2	176.6	25.7	502.5
	3	13.5	18.2	45.7
	4	47.8	41.6	290.8
	5	53.7	32.8	247.6
	6	26.7	1.6	41.7
	7	96.3	7.6	202.5
	8	40.2	192.1	69.3
	9	62.1	5.4	10.6
	10	90.8	12.6	185.0
	11	523.1	661.8	585.9
	12	138.8	179.4	148.7
	13	83.7	274.9	317.3
	14	98.6	10.7	10.6
	15	59.2	32.6	7.9
	16	307.5	100.0	48.3
	17	124.3	663.5	334.1
	18	95.7	11.2	192.6
	19	35.1	6.3	10.6
	20	159.2	1.0	146.1
	21	47.5	33.4	57.4
	22	199.4	77.6	106.6
	23	1.2	2.5	10.6
AIH4	1	1194.8	116.8	56.3
	2	14.4	3.3	10.6
	3	253.6	397.6	167.7
	4	424.7	193.2	67.5
	5	223.4	5.1	10.6
	6	264.3	19.6	140.5
	7	14.4	6.1	10.6
	8	4565.3	3861.2	33.3
	9	841.4	56.1	347.5
	10	0.8	44.0	10.6
	11	15990.1	1077.3	344.0
	12	20.2	3.6	10.6
	13	401.6	90.6	195.2
	14	1562.2	606.5	73.5
	15	6606.3	276.5	178.8
	16	2.5	5.5	10.6
	17	14.4	4.9	10.6
	18	20.6	4.0	10.6
	19	3652.3	173.2	67.3
	20	7751.3	273.1	452.3
	21	154.4	120.8	417.8

	22	25.6	7.0	10.6
	23	192.7	18.9	10.6
	24	1679.4	3254.3	10.6
AlH6	1	715.4	54.0	1854.3
	2	210.6	160.9	1588.4
	3	976.6	920.4	2482.3
	4	1.6	3.1	84.2
	5	158.5	16.9	848.4
	6	629.0	276.9	2134.9
	7	595.6	919.6	1554.5
	8	350.8	655.0	1298.8
	9	3.5	0.1	10.6
	10	3753.5	50.5	4600.8
	11	547.7	10.3	102.8
	12	7343.6	447.5	3332.3
	13	1874.2	19.3	2234.3
	14	1579.0	84.8	872.1
AlH8	1	47.7	596.2	36.1
	2	231.3	27.4	23.4
	3	162.7	5.4	34.7
	4	204.0	59.7	58.2
	5	112.8	4.3	22.8
	6	56.3	5.1	19.1
	7	10.9	984.0	353.9
	8	14.4	103.7	134.2
	9	144.5	27.0	26.1
	10	498.7	15.3	27.8
	11	96.9	5.4	29.2
	12	5.8	3.3	29.2
	13	1717.2	904.4	164.1
	14	54.3	100.4	39.4
	15	5715.2	139.2	32.7
	16	251.9	61.5	14.4
AlH9	1	73.1	5.0	634.0
	2	534.7	19.9	57.5
	3	13475.8	17.0	176.7
	4	14.9	299.7	195.6
	5	17.2	7.0	277.2
	6	3.0	13.7	21.5
	7	17.6	105.7	249.1
	8	26.1	273.6	579.2
	9	5359.2	7.2	260.8
	10	14.4	245.5	349.4
	11	34.3	15.3	462.8
	12	2571.9	2.5	130.1
	13	66.1	77.0	281.5
	14	2027.8	1.5	911.0
	15	12508.3	139.2	73.4
	16	1.0	8.4	10.8
	17	14.9	1.1	12.2
	18	8.4	80.7	268.3
	19	5.4	21.4	117.9
AlH10	1	75.7	0.2	2.6
	2	7.5	1.2	9.2
	3	320.2	2.5	29.8
	4	241.3	5.2	98.8
	5	1933.5	0.8	20.3
	6	2282.4	1659.9	1319.9
	7	1588.0	0.4	339.5
	8	890.1	0.1	72.0
	9	466.9	354.7	1014.3
	10	314.4	0.5	26.4
	11	52.1	0.2	5.7
	12	101.5	10.0	47.3
	13	1374.6	48.1	491.3
	14	1868.2	23.9	74.7

	15	14.6	0.1	3.7
	16	80.6	0.6	7.7
	17	3172.0	654.1	1164.9
	18	72.6	0.1	4.5
	19	14.4	0.7	6.1
	20	539.6	151.3	113.4
	21	2558.9	1.1	38.6
	22	45.6	0.4	50.5
	23	122.8	7.7	49.0
	24	251.3	1.1	10.8
	25	1413.4	922.3	626.7
	26	123.1	6.7	255.4
	27	883.5	886.1	454.3
AIH11	1	1079.1	1921.3	76.9
	2	147.7	1262.8	99.3
	3	788.6	46.3	109.4
	4	83.5	9.7	94.0
	5	4218.0	98.9	1022.2
	6	694.8	266.9	67.7
	7	446.5	31.3	288.4
	8	1105.1	248.4	69.8
	9	559.1	64.1	12.7
	10	579.1	192.2	19.6
	11	112.6	66.7	46.7
	12	156.3	251.1	40.3
	13	75.4	43.7	64.7
	14	3558.4	1564.8	945.0
	15	1329.6	789.7	548.1
	16	6079.3	3256.0	1337.7
	17	2140.7	1438.3	2042.8
	18	1309.0	178.8	1700.1
	19	4486.5	109.1	2814.5
	20	1368.9	490.2	5442.9
	21	5408.7	385.9	365.6
	22	140.9	489.0	2602.7
	23	464.9	37.5	685.9
	24	187.0	29.2	16.3
	25	2565.9	379.3	1181.9
	26	1709.0	1759.6	426.3
	27	215.6	264.9	36.1
	28	640.2	836.2	2876.7
	29	200.6	239.4	2538.4
	30	4719.3	42.0	22.9
	31	6296.9	3459.8	919.2
AIH12	1	0.1	0.1	3.4
	2	61.4	0.1	6.6
	3	0.1	0.1	4.8
	4	0.2	0.1	7.1
	5	15.6	0.1	7.1
	6	0.1	0.1	6.9
	7	0.1	0.1	4.1
	8	3118.1	5.9	22.7
	9	49.0	0.1	4.4
	10	0.1	0.1	3.9
	11	24.9	0.1	5.3
	12	10.7	0.3	9.2
	13	0.1	0.1	4.1
	14	14.9	0.1	6.2
	15	0.1	0.1	6.4
	16	42.4	0.1	4.1
	17	0.1	0.1	5.8
	18	190.8	0.1	10.0
	19	656.3	1.0	12.4
	20	174.5	18.7	28.0
	21	0.1	0.1	5.3
	22	0.1	0.1	6.2
	23	10.4	0.1	6.2

	24	0.1	0.1	1.8
	25	4316.5	1.3	228.4
	26	286.2	2.3	8.8
	27	0.2	0.2	8.8
	28	63.6	0.5	8.4
	29	0.1	0.1	5.8
	30	0.1	0.1	3.4
AIH13	1	14.4	4.7	10.6
	2	14.4	2.5	10.6
	3	3.5	3.5	10.6
	4	14.4	3.6	10.6
	5	29.9	2.2	10.6
AIH14	1	15.0	44.7	0.1
	2	1.0	1.0	0.1
	3	1.8	1.0	0.1
	4	1.0	1.0	0.1
	5	1.0	1.0	0.1
	6	46.9	2.8	0.1
	7	1.0	1.0	0.1
	8	1.0	1.0	0.1
	9	1.0	1.0	0.1
	10	1.0	1.0	0.1
	11	1.0	1.0	0.1
	12	1.0	1.0	0.1
	13	1.0	1.0	0.1
	14	1.0	1.0	0.1
	15	35.6	1.0	0.1
	16	2.2	1.0	0.1
	17	1.0	1.0	0.1
	18	1.0	1.0	0.1
	19	44.8	43.3	0.1
	20	1.0	1.0	0.1
	21	7.6	80.9	0.1
	22	1.0	1.0	0.1
	23	65.2	37.0	0.1
	24	1.0	1.0	0.1
	25	14.4	1.0	3.3
	26	5.6	68.2	82.6
	27	277.9	2.2	4.5
	28	33.0	13.8	4.9
	29	11.3	13.8	16.1
	30	525.9	619.9	166.6
	31	17967.0	325.3	729.0
	32	4191.0	33.3	883.4
	33	331.6	134.9	923.7
	34	1085.3	703.2	883.1
	35	8.9	219.5	90.6
	36	181.5	108.4	84.8
	37	193.2	599.4	77.7
	38	3211.0	3092.9	898.5
	39	23.7	1.1	3.3
	40	1808.4	3.8	25.6
	41	1648.2	3.9	47.8
	42	130.0	37.0	94.5

ID	Clone n.	IFN-γ (pg/ml)	IL-10 (pg/ml)	IL-4 (pg/ml)
PC1	1	51.8	47.6	119.6
	2	29.2	1.1	0.1
	3	31.8	169.0	0.1
	4	0.1	7.0	2.9
	5	74.6	11.0	2.1
	6	385.3	384.9	7.5
	7	335.5	24.8	0.1
	8	296.7	63.3	0.3

	9	3.4	50.4	170.8
	10	245.0	47.1	6.6
	11	4513.3	69.4	287.4
	12	4629.5	76.8	5.3
	13	1209.0	8.1	143.9
	14	9.0	316.8	81.2
	15	5608.7	112.0	131.1
	16	0.1	12.3	0.6
	17	4354.4	165.5	86.2
	18	30.9	26.8	3.4
	19	22.7	37.6	45.7
	20	6793.2	271.7	43.4
	21	621.7	14.2	3.4
	22	895.0	535.4	125.5
	23	25.0	2.2	0.6
	24	6054.4	44.1	180.6
PC3	1	3.5	3.0	10.6
	2	5656.1	8.2	18.4
	3	2276.9	8.0	10.6
	4	2862.3	138.1	107.0
	5	5376.1	39.8	141.1
	6	12894.5	92.2	82.2
	7	3742.4	57.9	214.5
	8	4605.0	24.4	42.7
	9	536.4	5.3	10.6
	10	458.3	3.6	10.6
PC5	1	14.4	1.9	174.1
	2	3320.7	2386.7	287.7
	3	14.4	0.3	107.1
	4	5637.4	2323.5	542.5
	5	8840.9	2308.9	658.0
	6	1974.7	696.6	238.9
	7	83.2	470.4	358.2
	8	5981.9	438.1	219.4
	9	64.5	792.4	63.5

ID	Clone n.	Clonotype	IFN-γ (pg/ml)	IL-10 (pg/ml)	IL-4 (pg/ml)
AIH12 LB	1	2	1802.8	866.4	1784.9
	2	2	1133.2	154.4	3746.2
	3	2	750.6	41.2	3994.6
	4	2	1459.0	799.9	3881.0
	5	2	701.4	323.8	2703.0
	6	2	770.1	524.6	1508.5
	7	2	944.2	558.4	1956.1
	8	2	638.6	171.3	2408.6
	9	2	729.8	160.2	1035.9
	10	2	1367.5	371.6	9487.0
	11	2	1054.0	171.3	4187.4
	12	2	624.9	123.8	3539.1
	13	2	1835.6	185.2	6799.7
	14	2	357.0	54.0	3267.7
	15	2	1742.5	96.9	5405.2
	16	2	701.0	41.2	4095.7
	17	2	474.1	5.0	6994.5
	18	2	1093.3	160.2	2343.0
	19	2	291.5	88.6	2216.5
	20	2	650.6	219.2	5114.5
	21	2	755.9	239.2	4419.6
	22	2	1035.2	232.7	6498.6
	23	2	886.4	369.9	3190.5
	24	3	172.5	91.4	1480.1
	25	3	282.2	127.6	5502.1
	26	3	309.5	88.6	3286.9
	27	3	183.5	63.8	2332.8

Supplemental Table 6. Primers for BCR and TRVB sequencing

BCR	
RT-PCR (constant region primers)	HuIgG-const-anti: 5'-TCTTGTCCACCTTGGTGTTGCT-3'<br>Hu-CK-for: 5'- ACACTCTCCCCTGTTGAAGCTCTT-3'<br>Clambda-as296: 5'-ACTGTCTTCTCCACGGTGCT-3'
VH primer mix:<br>Reverse (constant region primer)<br><br>Forward (variable region primers)	 IGHG1-4 Rev: 5'-ACGGTCACCACGCTGCTGAG-3'<br><br>VHL1: 5'- TCACCATGGACTGSACCTGGA-3'<br>VHL1_58: 5'-ATGGACTGGATTTGGAGG-3'<br>VHL2: 5'-ATGGACACACTTTGCTMCAC-3'<br>VHL2b: 5'-ATGGACATACTTTGTTCCAC-3'<br>VHL3bis: 5'-TCACCATGGAGTTKGGGCTGAGC-3'<br>VHL3-21: 5'-TCACCATGGAACTGGGGCTCCGC-3'<br>VHL3-64-01: 5'-ATGACGGAGTTTGGGCTGAGC-3'<br>VHL4: 5'-AGAACATGAAACAYCTGTGGTTCTT-3'<br>VHL5: 5'-ATGGGGTCAACCGCCATCCT-3'<br>VHL6: 5'-CAATGTCTGTCTCCTTCCTCATC-3'<br>VHL7: 5'-ATGGACTGGACCTGGAGGATCC -3'
VK primer mix:<br>Reverse (constant region primer)<br><br>Forward (variable region primers)	 Ckappa_as266 (KasD): 5'-GACTTCGCAGGCGTAGACTT-3'<br><br>VKL1bis: 5'-GCTCAGCTCCTGGGGCTYCTG-3'<br>VKL2: 5'-CTGGGGCTGCTAATGCTCTGG-3'<br>VKL3: 5'-TTCCTCCTGCTACTCTGGCTC-3'<br>VKL4: 5'-CAGACCCAGGTCTTCATTTCT-3'<br>VKL5: 5'-TCCCAGGTTCACCTCCTCAGC-3'<br>VKL6: 5'-TTCTGCTSCTCTGGGTTCCA-3'
VL primer mix:<br>Reverse (constant region primer)<br><br>Forward (variable region primers):	 3'CL: 5'-CACYAGTGTGGCCTTGTTGGCTTG-3'<br><br>VLL1bis: 5'-CCTCTCCTCCTCACYCTCCT-3'<br>VLL2bis: 5'-ATGGCCTGGGCTCTGCTGCTC-3'<br>VLL3a: 5'-ATGGCCTGGACCCCTCTC-3'<br>VLL3b: 5'-ATGGCCTGGATCCCTCTM-3'<br>VLL3-9/21: 5'-ATGGCCTGGACCGYTCTC-3'<br>VLL3-22: 5'-ATGGCATGGGCCACACTCC-3'<br>VLL4: 5'-TGGGTCTCCTTCTACCTAC-3'<br>VLL4a: 5'-TCCTCCTCCACTGCACAGG-3'<br>VLL5-7-9: 5'-ATGGCCTGGRCTCCTCTS-3'<br>VLL8: 5'-ATGGCCTGGATGATGCTTC-3'<br>VLL10: 5'-TCACTCACTCTGCAGTGTC-3'

TRBV	
Reverse (TRBC-rev84)<br><br>Forward (Maxipool FRW mix)	5'-TGTGGCCTTTTGGGTGTGG-3'<br><br>5'-TCAGGTGTGATYCAATTTC-3'<br>5'-AGGTGTGATCCAATTTCG-3'<br>5'-TGTGTCCTGGTACCAACAG-3'<br>5'-GTATCGACAAGACCCAGG-3'<br>5'-GTATCGACAAGACCYGGG-3'<br>5'-CTCACCTGAATGCCCCAA-3'<br>5'-ATGTTYTGGTAYCGTCAG-3'<br>5'-CCTTTACTGGTACCDGCAGA-3'<br>5'-ACAGAGATGGGACAAGAAG-3'<br>5'-GCCATGTACTGGTAYMGA-3'<br>5'-CCCCATCTCTAATCACTTATAC-3'<br>5'-ACATCAAACCCCAACCTATAC-3'<br>5'-ACCAGCAGAAGTCAAGTCA-3'<br>5'-TGTSTACTGGTACCARCAG-3'<br>5'-GGGAAGGACAGAAAGCAAAA-3'<br>5'-TTACTCAGTTCCCCAGCC-3'<br>5'-AGATGCAGCCCAATGAAA-3'<br>ACAGATGGGAAACGACAA-3'<br>GTATCRACAAGAYCCAGGA-3'
